# Single-nucleus transcriptome inventory of giant panda reveals cellular basis for fitness optimization under low metabolism

**DOI:** 10.1186/s12915-023-01691-2

**Published:** 2023-10-20

**Authors:** Shangchen Yang, Tianming Lan, Rongping Wei, Ling Zhang, Lin Lin, Hanyu Du, Yunting Huang, Guiquan Zhang, Shan Huang, Minhui Shi, Chengdong Wang, Qing Wang, Rengui Li, Lei Han, Dan Tang, Haimeng Li, Hemin Zhang, Jie Cui, Haorong Lu, Jinrong Huang, Yonglun Luo, Desheng Li, Qiu-Hong Wan, Huan Liu, Sheng-Guo Fang

**Affiliations:** 1 MOE Key Laboratory of Biosystems Homeostasis & Protection, State Conservation Centre for Gene Resources of Endangered Wildlife, College of Life Sciences, Zhejiang University, Hangzhou, 310058 China; 2 State Key Laboratory of Agricultural Genomics, BGI-Shenzhen, Shenzhen, 518083 China; 3 BGI Life Science Joint Research Center, Northeast Forestry University, Harbin, 150040 China; 4 Key Laboratory of State Forestry and Grassland Administration (State Park Administration) on Conservation Biology of Rare Animals in the Giant Panda National Park, China Conservation and Research Center for the Giant Panda, Dujiangyan, 611830 China; 5 China Wildlife Conservation Association, Beijing, 100714 China; 6 Department of Biomedicine, Aarhus University, 8000 Aarhus, Denmark; 7 Lars Bolund Institute of Regenerative Medicine, Qingdao-Europe Advanced Institute for Life Sciences, BGI-Qingdao, Qingdao, 266555 China; 8 Steno Diabetes Center Aarhus, Aarhus University Hospital, 8000 Aarhus, Denmark; 9 China National GeneBank, BGI-Shenzhen, Shenzhen, 518120 China; 10 College of Life Sciences, University of Chinese Academy of Sciences, Beijing, 100049 China; 11 College of Wildlife Resources, Northeast Forestry University, Harbin, 150040 China; 12 The Genome Synthesis and Editing Platform, BGI-Shenzhen, Shenzhen, 518120 China; 13 Guangdong Provincial Key Laboratory of Genome Read and Write, BGI-Shenzhen, Shenzhen, 518120 China; 14 BGI-Shenzhen, Shenzhen, 518083 China

**Keywords:** Single-nucleus transcriptome atlas, Giant panda, Low metabolism, Energy homeostasis, Adaptation

## Abstract

**Background:**

Energy homeostasis is essential for the adaptation of animals to their environment and some wild animals keep low metabolism adaptive to their low-nutrient dietary supply. Giant panda is such a typical low-metabolic mammal exhibiting species specialization of extremely low daily energy expenditure. It has low levels of basal metabolic rate, thyroid hormone, and physical activities, whereas the cellular bases of its low metabolic adaptation remain rarely explored.

**Results:**

In this study, we generate a single-nucleus transcriptome atlas of 21 organs/tissues from a female giant panda. We focused on the central metabolic organ (liver) and dissected cellular metabolic status by cross-species comparison. Adaptive expression mode (i.e., AMPK related) was prominently displayed in the hepatocyte of giant panda. In the highest energy-consuming organ, the heart, we found a possibly optimized utilization of fatty acid. Detailed cell subtype annotation of endothelial cells showed the uterine-specific deficiency of blood vascular subclasses, indicating a potential adaptation for a low reproductive energy expenditure.

**Conclusions:**

Our findings shed light on the possible cellular basis and transcriptomic regulatory clues for the low metabolism in giant pandas and helped to understand physiological adaptation response to nutrient stress.

**Supplementary Information:**

The online version contains supplementary material available at 10.1186/s12915-023-01691-2.

## Background

Optimization of the interplay between energy metabolism and biological production (growth and reproduction) is characteristic of life and can maximize organisms’ fitness (i.e., lifetime reproduction) and adaptation to their environment [1]. Energy homeostasis tightly relies on cellular metabolic regulation to maintain a balance between food intake and energy expenditure, which begins with nutrition sensing [2]. The powerful and sophisticated mechanisms in cells are products of evolution, which help to regulate metabolic processes towards cellular energy status [3]. Two key sensors, AMP-activated protein kinase (AMPK) and target-of-rapamycin (TOR), antagonistically play a central role in switching on the catabolism in nutrient starvation or anabolism in nutrient availability. Phosphorylated AMPK can also inhibit cell growth and promote autophagy and lysosome biogenesis to recycle cytoplasmic components for essential cellular activities [4]. However, its counterpart, TOR complexes 1 and 2 (TORC1 and TORC2), can inhibit the activation of AMPK and promote contrary pathways (cellular anabolism and cell growth) [3]. The two hubs in the lysosomes/vacuoles represent a conserved mechanism to regulate metabolism and safeguard the energy balance in eukaryotes [5, 6].

Metabolic allometries are observed at wide scales including enzymes, mitochondria, cells, whole organisms, and even ecosystems [7]. It is now regarded as an optimization rather than a constraint on an energy-expenditure budget [1]. The heart is the most energy-demanding organ in the body for contractile function. It has the flexibility to use different substrates for ATP generation. Healthy adult cardiomyocytes (CMs) preferentially utilize fatty acids (FAs) as an energy substrate [8–10]. In adult fasting mammals, fatty acid oxidation (FAO) supplies for 60–80% of cardiac energy metabolism with the remainder provided by glucose, lactate, and ketone metabolism [11]. Lipids for the heart are mainly the circulating esterified FAs bound to lipoproteins (TGRLPs, triglyceride-rich lipoproteins) from the liver and slightly non-esterified (free) fatty acids (NEFAs) from adipose tissues [12, 13]. CMs also have capacities on lipoprotein formation and secretion to prevent lipotoxicity of TAG and FA accumulation [14]. Elevated triacylglycerol synthesis and FA β-oxidation were observed to be novel signatures of longevity in healthy aging [15].

The circulating system is vital for nutrient delivery across the body. The portal vein from the gastrointestinal tract to the liver, also known as the gut-liver axis, directly provides an anatomic channel for dietary substance uptake in the liver [16]. Then, the liver-heart axis plays a crucial role in cardiac substrate supply and liver lipid homeostasis [17, 18]. Endothelial cells (ECs) line the inner layer (tunic intima) of blood vessels, serving as a barrier to deliver nutrients and maintain microenvironment homeostasis. Cellular heterogeneity of ECs could reflect diverse transmembrane transport and metabolic functions serving for specific tissues across vascular beds (artery, venule, capillary, etc.) [19]. Lipoprotein lipase (LPL) is a key enzyme for the distribution of circulating lipids across organs [8]. Glucose transporters were expressed in tissue-specific ECs (i.e., brain, testis), implying different nutrient requirements throughout the whole body [19]. Interrogation of EC heterogeneity helps to study the nutritional support for distinct local organs/tissues.

The giant panda, *Ailuropoda melanoleuca*, is a flagship species in the global biodiversity conservation and enjoys an iconic status in studying the survival and adaption of wildlife. It was once highly endangered and aroused a heated debate about whether this species is an evolutionary cul-de-sac, given that its unique biological and physiological characteristics seemed to put it on the edge of extinction vortex [20–22]. Giant panda evolves from the family of *Ursidae* and nowadays it lives on a bamboo-dominated diet, which contains limited nutrients. What’s worse, they still keep a typical carnivorous gastrointestinal tract, leading to a very low digestive efficiency. Nevertheless, amazingly, their daily energy expenditure was also exceptionally low (37.7% of the mammalian expectation), which was partly attributed by few daily activities, low body temperature, reduced inner organ sizes, and low reproduction rate [23, 24]. The quality and quantity of their food are regarded as the ultimate factors of their low metabolism [23]. It has been measured that the liver was the most reduced organ in giant panda, 62.8% of the expected size, followed by kidney (74.5%) and brain (87.5%) while the size of its heart increased (104.9%) [23]. Energy used for reproduction also seems inadequate that giant panda cubs are poorly developed and the ratio of cub/mother body weight is about 1/900th, which hits the lowest among all Eutherians [22, 24]. All the aforementioned biological specializations make the giant panda a typical and ideal case to explore the cellular basis of natural optimization of energy utilization under a low basal metabolic rate.

With high-throughput single-cell and single-nucleus RNA sequencing (scRNA-seq and snRNA-seq, respectively) technologies, it is accessible to dissect cellular heterogeneity and monitor gene expression patterns in single cells/nuclei, getting closer to the cellular organization and regulation for fitness optimization under low metabolism in giant panda. Here, we conducted large-scale single-nucleus sequencing in a giant panda and constructed a snRNA-seq atlas of 21 organs/tissues from one female donor. We firstly presented the cellular transcriptome profiling of giant panda and made a cross-species comparison, indicating that hepatocyte was remarkably different in giant panda. By focusing on genes involved in AMPK and TOR signaling pathways, we proposed a possible cellular basis of low metabolism and downstream affected processes. Secondly, the cooperation of cardiomyocytes on fatty acid oxidation for ATP generation was discovered in the largest energy-consumed organ (heart). Thirdly, the nutrient delivery network in all studied organs/tissues was characterized from the perspective of ECs. In principle, our data help to answer previously unsolved questions of giant pandas at a single-nucleus resolution, serving as a powerful foundation and resource for future studies on evolution and adaption.

## Results

### Single-nucleus transcriptome landscape of 21 organs/tissues of the giant panda

**Fig. 1:**
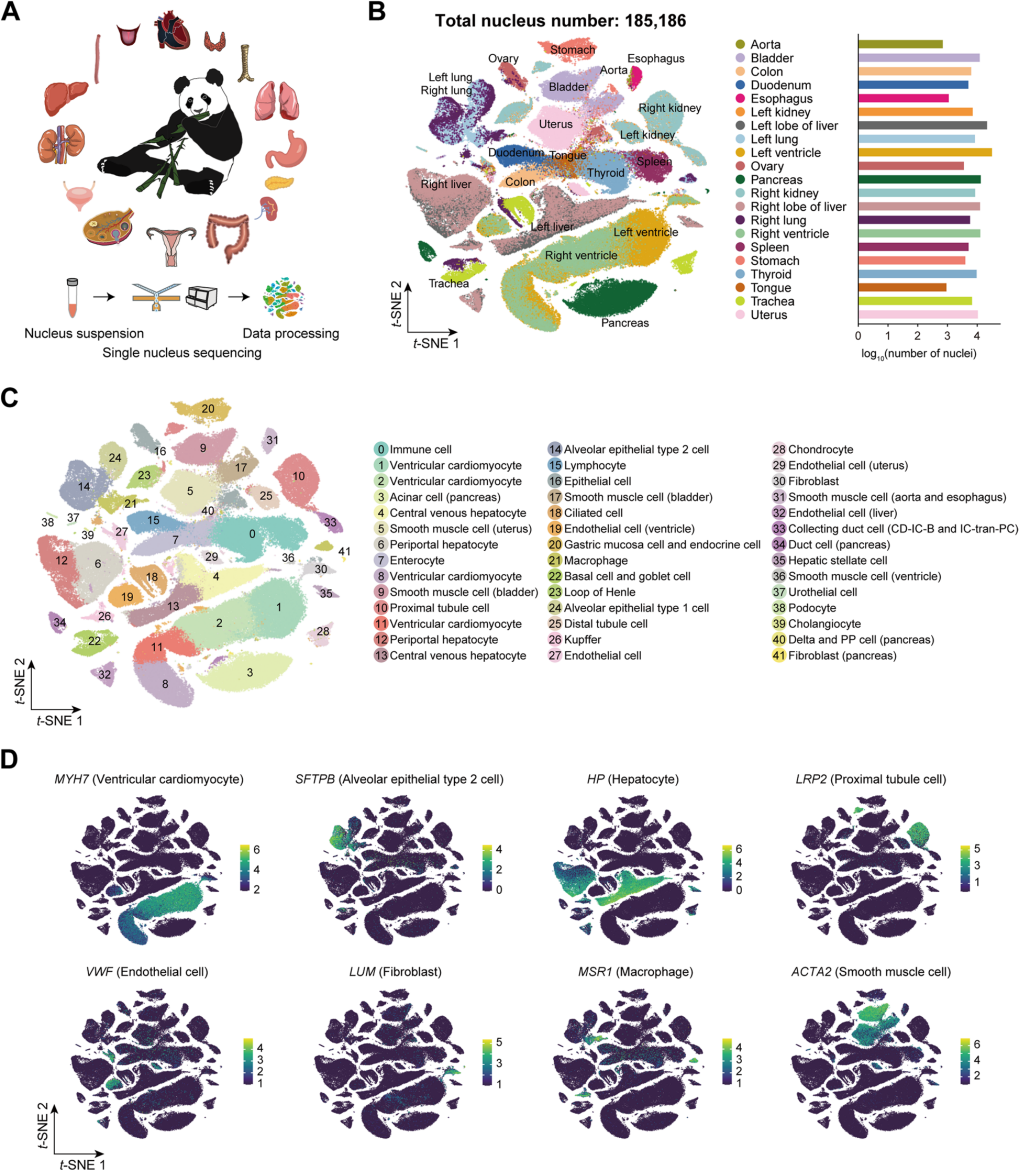
Landscape of the snRNA-seq atlas of giant panda. **A** Schematic representation of 21 organs/tissues collected from a female donor with the main experimental and analysis procedures underneath. **B** *t*-SNE visualization of all nuclei colored by organ/tissue (left) and number of nuclei from each organ/tissue passing quality control (right). **C** *t*-SNE visualization of cell types labeled by different colors. **D** *t*-SNE map showing the expression of classical marker genes in some tissue-specific or common cell types. *n* = 185,186 single nucleus

To characterize the transcriptome landscape of the giant panda at a single-cell resolution, we conducted single-nucleus RNA sequencing of 21 major organs/tissues from a female donor (Fig. 1A; Additional file 1: Fig. S1). A total of 41.86×10^9^ RNA reads were obtained, with counts ranging from 9.73×10^8^ for the spleen to 3.15×10^9^ for the left lobe of the liver (Additional file 2: Table S1). After filtering doublets and low-quality nuclei (see the “Methods” section), we finally retained 185,186 nuclei with an average of 977 unique molecular identifiers (UMIs) and 572 genes per nucleus (Additional file 1: Fig. S2A, B; Additional file 2: Table S2). The number of UMI counts and genes detected varied slightly across organs/tissues. Nuclei of the pancreas showed the highest median counts (1466 UMIs and 871 genes per nucleus), but only 474 UMIs and 300 genes per nucleus were recovered for the esophagus. In detail, we captured nuclei from seven systems: cardiovascular system, 44,307 nuclei (aorta, 692; left ventricle, 31,045; and right ventricle, 12,570); respiratory system, 20,606 nuclei (left lung, 8261; right lung, 5694; and trachea, 6651); digestive system, 51,112 nuclei (left liver, 21,429; right liver, 12,419; colon, 6241; duodenum, 5032; stomach, 3974; esophagus, 1097; and tongue, 920); urinary system, 27,553 nuclei (left kidney, 7049; right kidney, 8494; and bladder, 12,010); endocrine system, 22,526 nuclei (pancreas, 13,089; thyroid, 9437); immune system (spleen), 5112 nuclei; and reproductive system, 13,970 nuclei (ovary, 3564; uterus, 10,406) (Fig. 1B).

We next annotated each cluster according to their transcriptome signatures. To guarantee the correctness of cell identity annotation, we used unsupervised clustering to annotate the nucleus for both each single tissue (Additional file 1: Fig. S3–5) and 21 tissues as a whole (Fig. 1C, D; Additional file 1: Fig. S6). By combing the two annotation results, we finally identified 46 major cell types in the 21 tissues (Additional file 2: Tables S3, 4) [19, 25–43], with one cell type in tongue as the least and twelve cell types in the left kidney as the most (Additional file 2: Table S2). Commonly shared cell types were achieved among these tissues, such as endothelial cell (EC), fibroblast (FB), macrophage (MP), and smooth muscle cell (SMC), which was consistent with previous studies [25, 44]. Organ-specific cell types were also captured, with the top three sources being kidneys (six: proximal tubule cell (PTC), distal tubule cell (DTC), loop of Henle (LOH), podocyte (POD) and collecting duct intercalated cell - type B (CD-IC-B)), intercalated cell transiting to principal cell (CD-tran-PC), livers (four: hepatocyte, hepatic stellate cell (HSC), Kupffer and cholangiocyte) and pancreas (four: acinar cell, duct cell, delta cell and PP cell). Lastly, we found some cycling cells in this old panda with their representative populations in the lungs (cycling alveolar epithelial type 2 (AT2) and cycling MP) and stomach (cycling pit mucosa cell (PMC)), implying that at least these cells were active and might have proliferative potential.

In the global landscape, clustering analysis identified 42 clusters with the nucleus counts ranging from 230 to 14,262 per cluster (Additional file 1: Fig. S7A). Global cell annotation was highly consistent with the result of the single-organ atlas (Additional file 1: Fig. S7B–D). However, some cells were mixed with other large cell populations, which we suspected as clustering errors on a global scale. Based on the global clustering and cell identities from a single organ atlas, we explored their clustering performance across the whole body. In the global atlas, all nuclei tended to group together on the basis of cell type and organ origin, exhibiting the similarity of their expression profiles. For paired organs (such as ventricles, lungs, livers, and kidneys), their cells coincided with the same cell type as their counterparts, while the same types from collaborative and adjacent organs (such as basal cell, goblet cell, and ciliated cell of lungs and trachea) just bordered by each other (Fig. 1B; Additional file 1: Fig. S7E). Common cell types across different tissues were mixed in certain clusters, such as ECs (cluster 27), FBs, and MPs, showing their similar expression patterns. There were also a number of cells clustered based on tissue origins, showing the heterogeneity of specific cell types and possibly different functions serving for these tissues [19].

### Cellular response to low-nutrient diet in giant panda

We next sought to gain better insights into the cellular characteristics of the low basal metabolism of the giant panda. To this end, we performed cross-species comparison with single-cell/nucleus atlases from humans (Human Cell Landscape, HCL) [25, 45], crab-eating monkeys (Non-Human Primate Cell Atlas, NHPCA) [46, 47], and mouse (Mouse Cell Atlas, MCA) [48, 49], reasoning that cross-species comparison enables us to uncover conserved and divergent features in giant panda. At a first glance, integration of sc-RNA/sn-RNA data from the four species showed a good correlation in the single-organ atlas (except liver) (Additional file 1: Fig. S8). Next, based on previous cell identities and differential marker genes [46], we examined the existence of shared cell types and the potential emergence of species-specific cell populations. Our cell type annotation results are consistent with those from published studies (Additional file 1: Fig. S9), suggesting a similar and conserved cellular repertoire between giant pandas and other three species.

**Fig. 2:**
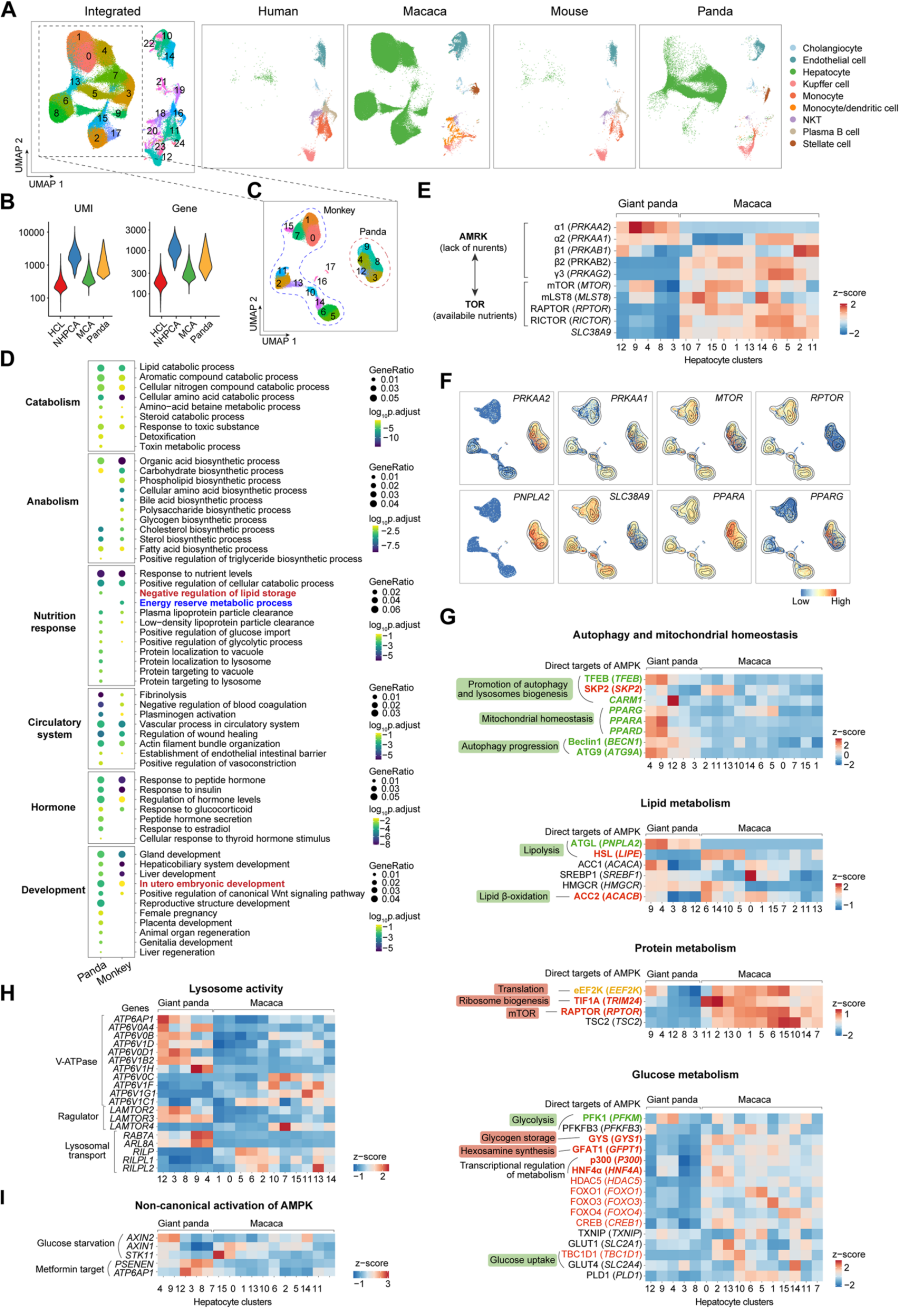
Transcriptomic divergence of hepatocyte revealed by cross-species comparison. **A** UMAP representation of four species comparison among human, monkey, mouse, and giant panda datasets of liver. Cell identity was in the right legend. **B** Violin plots indicating the numbers of UMI and genes detected in liver datasets of human, monkey, mouse, and giant panda. **C** Re-clustering of all hepatocyte clusters from (**A**) and circled by species. **D** GO enrichment analysis for DEGs of hepatocytes from monkey and giant panda. **E** Expression level of AMPK and TOR subunits in each hepatocyte cluster. **F** UMAP representations with nuclei colored by the expression level of essential genes. **G** Promoted or repressed metabolic signal pathways which were direct downstream targets of activated AMPK. **H** Expression level of genes involved in lysosome activity. **I** Potential non-canonical activation of AMPK

Firstly, in the most vital metabolic organ, the liver, the whole landscape consisted of several hepatocyte populations, HSC, EC, and immune cells (clustered together regardless of species source) (Fig. 2A). Here, UMI and gene numbers of the giant panda were higher than HCL and MCA but lower than NHPCA (Fig. 2B). Due to the same sn-RNA method applied to the monkey and the giant panda, the abundance of hepatocyte was increased, offering opportunities to investigate potential gene expression specialization. Re-clustering of hepatocytes exhibited a separation based on species difference (Fig. 2C), implying transcriptomic divergence of different species. Gene ontology (GO) analysis for differentially expressed genes (DEGs) showed conserved functions in the monkey and the giant panda (Fig. 2D), such as lipid catabolic process, cellular nitrogen compound catabolic process, and cholesterol biosynthetic process, which was consistent with the physiological functions of the liver on dietary digestion. There were several anabolic processes significantly presented in the monkey while absent in the giant panda, including phospholipid, amino acid, bile acid, and glycogen biosynthesis. And the undetected bile acid-related term in giant pandas was consistent with previous bulk-RNA research [50], due to the low-fat diet. Metabolism regulation differences could be reflected by distinct enriched GOs related to nutrition response: “negative regulation of lipid storage” in giant pandas versus “energy reserve metabolic process” in monkeys. Such different cellular energy statuses could also be further implied by relevant metabolic characteristics: glucose import was positively regulated and lysosome/vacuole activities were active in giant pandas, which was in line with the AMPK activation status. For other vital functions of the liver, they both show a connection with the circulatory system, hormone response, and development of tissues. The putative relationship with reproductive structure and embryonic development shed insights on cellular regulation for nutrition supply to reproductive organs.

As related to the environmental adaptation of giant pandas, a detailed comparison was next focused on pathways tightly related to nutrient-sensing proteins. We mapped the genes encoding AMPK and TOR proteins and found that AMPK α1 and α2 were upregulated in the giant panda whereas subunits of TOR were upregulated in the monkey, together with its catalyzer (*SLC38A9*), a lysosomal transmembrane protein sensing amino acid arginine and activating mTORC1 [51] (Fig. 2E, F). By characterizing the expression of direct downstream targets by AMPK, we discovered consistent signatures for low metabolism in giant pandas (Fig. 2G). Autophagy and mitochondrial homeostasis were notably promoted by overexpression of *TFEB*, *CARM1*, *PPARA*, *PPARD*, *BECN1*, and *ATG9A* [3, 52]. As the organelle of autophagy, lysosome also had an active activity through higher expression of V-ATPase and its regulator (Fig. 2H). For lipid, protein, and glucose metabolism, targeted genes promoting lipolysis, lipid β-oxidation, and glycolysis were upregulated while translation, ribosome biogenesis, mTOR, glycogen storage, and hexosamine synthesis were repressed in giant panda. In addition, upstream genes activated AMPK including *AXIN2*, *PSENEN*, and *ATP6AP1* were overexpressed in giant panda hepatocytes (Fig. 2I).

Lastly, hepatocytes in the giant panda were significantly increased in the toxin metabolic process and amino-acid betaine metabolic process (Fig. 2D), which were abundant in bamboo leaves [53]. Regarding the secondary metabolites in bamboo, we also mapped the well-known CYP450 family. They were mainly enriched in hepatocytes, together with HSC and cholangiocytes (Additional file 1: Fig. S10A). The main bitter substance in bamboo shoots is L-phenylalanine [54] and can be decomposed by phenylalanine-4-hydroxylase (encoded by *PAH*) in liver and kidney [55]. We discovered that *PAH* was predominantly expressed in hepatocytes (65.49%), HSC (42.24%), cholangiocyte (36.81%), Kupffer (26.22%), and EC (20.72%) in livers and the proximal tubule cell (19.73%) in kidneys (Additional file 1: Fig. S10B). These features collectively provided the first single-cell transcriptome insights into the metabolic rewiring of giant panda hepatocytes’ adaptation to their environment.

### Cellular cooperation of vCMs in the largest energy-consuming organ

**Fig. 3:**
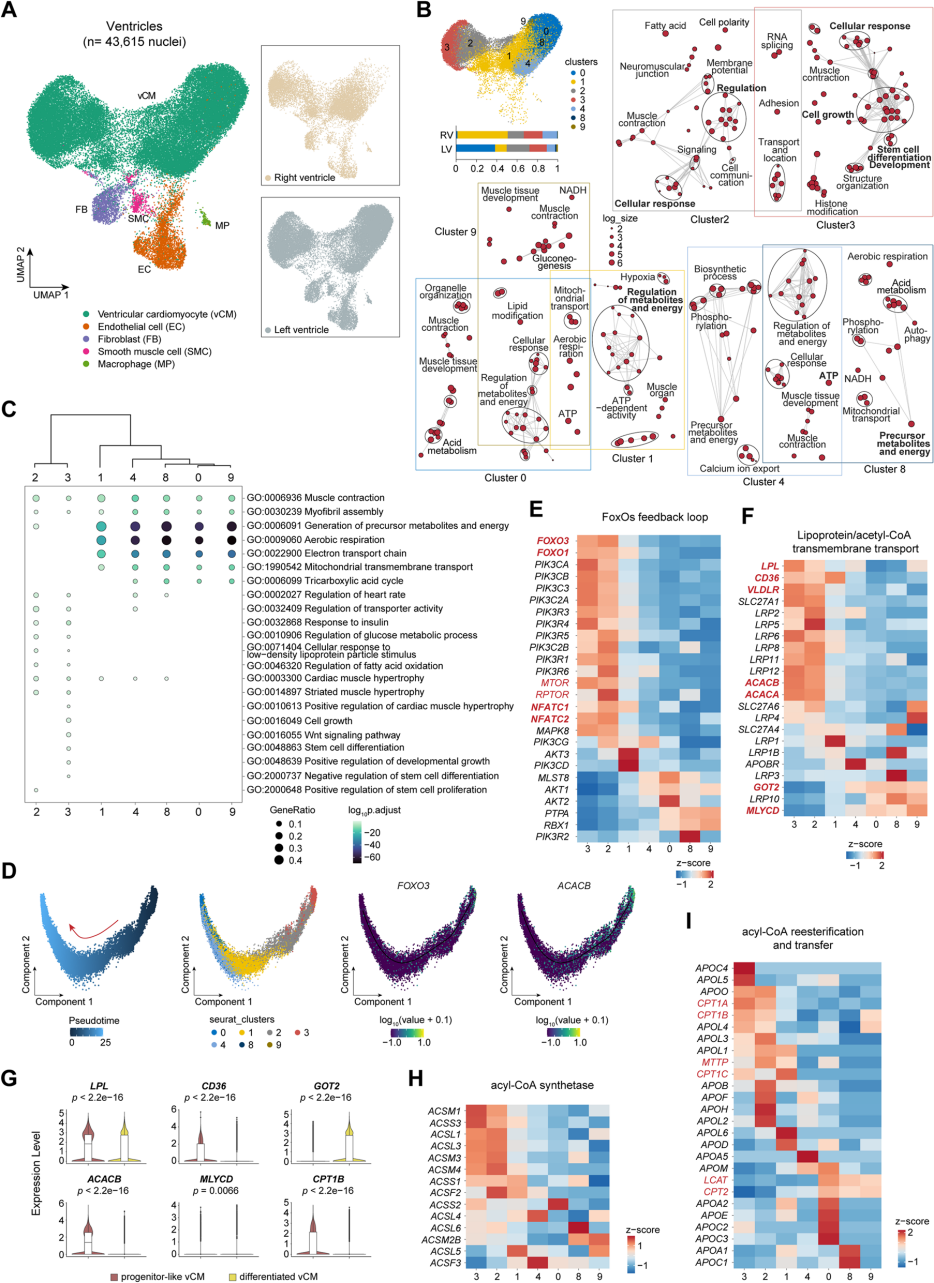
Cell profiles of ventricles and metabolic differences among vCMs. **A** UMAP visualization of ventricles and left and right counterparts. **B** Cell fraction of seven vCM clusters in left and right ventricles and GO enrichment analysis examining shared and distinct functions for each vCM cluster. **C** Function differences between clusters 2 and 3 and clusters 0, 1, 4, 8, and 9, with hierarchical clustering dendrogram of gene expression above them. **D** Monocle 2 pseudotime-ordered trajectory of vCMs. **E** Expression difference of FoxO TFs and their feedback loop involving in cardiac muscle remodeling between two vCM subtypes. **F** Transcriptomic comparison of genes responsible for lipoprotein uptake and oxidation in two vCM subtypes. **G** Violin plots showed a significant expressional difference of essential genes for the FA metabolic process. **H** Expression of genes encoding acyl-CoA synthetase. **I** Examination of genes for re-esterification of fatty acyl CoA and export

We next investigated the cardiac metabolic adaptations in the giant panda, with a focus on the cardiomyocytes. A total of 43,615 nuclei from the left and right ventricles of the heart were captured, representing five major cell types in 11 clusters, including ventricular cardiomyocyte (vCM), FB, EC, SMC, and MP (Fig. 3A). Seven clusters (c0, c1, c2, c3, c4, c8, and c9) were identified as vCMs based on the markers they specifically expressed (*TNNI3*, *MYH7*, *MYL2*, and *MYL3*), and GO terms suggested their common functions on muscle contraction, cardiac muscle tissue development, myofibril assembly and heart process (Fig. 3B, C), which were similar to previous human cardiac single cell/nucleus atlas [29, 32]. The vCM heterogeneity was characterized by remarkable differences in other putative functions: (1) c0, c1, c4, c8, and c9 intended to have high ATP-generating capacity by mitochondria, considering their major performances on the generation of precursor metabolites and energy, aerobic respiration, electron transport chain, and tricarboxylic acid cycle. Their marker genes also included nuclear-encoded mitochondrial genes, which were previously reported in the small high energetic population (vCM4) of humans [29]; (2) c2 and c3 seemed to resemble sensors and regulators with overexpressed genes related to cellular response to low-density lipoprotein particle stimulus, fatty acid and insulin stimulus, regulation of heart rate, transporter activity, fatty acid oxidation and glucose metabolic process, additionally, involving in cardiac muscle hypertrophy. And, c1 was relatively deficient in mitochondrial activities compared to c0, c4, c8, and c9 (Fig. 3C). The remarkably different cellular fractions of c0 and c1 in left and right counterparts (c0: 38.47% of left vCMs while 1.36% of right vCMs; c1: 11.33% of the left while 49.50% of right) (Fig. 3B), may explain the cellular basis supporting systemic and pulmonary circulation, respectively.

GO enrichment also implied that c3 was significantly related to cell growth, negative regulation of stem cell differentiation, and *Wnt* signaling pathway and c2 was involved in positive regulation of stem cell proliferation. And pseusdotime analysis supported the trajectory of vCM maturation from c3 to c2 and then to other clusters (Fig. 3D). So here we roughly divided all vCMs into two subclasses: progenitor-like (c3 and c2) and differentiated cells (c0, c1, c4, c8, and c9). We noted the well-known transcription factors (TFs), *FOXO3* and *FOXO1*, were mainly expressed in progenitor-like vCMs (Fig. 3E). Feedback loop of FoxO TFs is of crucial roles in modulating cardiomyocyte growth and survival [56]. We observed related phosphatidylinositol 3-kinase (PI3K), mTORC1, and nuclear factor of activated T cell 1 (NFAT1) were also overexpressed in the same subtypes, may be responsible for cardiac muscle remodeling in giant pandas.

Key genes involved in fatty acid intake in cardiomyocytes were studied, which were important for ATP generation in mitochondria. As expected, fatty acid-binding proteins (FABPs) and fatty acid transporters (FATPs) were widely expressed across vCMs. However, they showed a bias on the transmembrane transport efficiency of TGRLP or NEFA. Lipoprotein lipase (*LPL*), fatty acid transposase (*CD36*), and very low-density lipoprotein receptor (*VLDLR*) were mainly expressed in progenitor-like vCMs while differentiated ones highly expressed plasma membrane-associated fatty acid-binding protein (FABPpm, *GOT2*), which had a high affinity with NEFA (Fig. 3F, G). Furthermore, they would both be the FA-activated places that expressed acyl-CoA synthetase (Fig. 3H). However, they had an opposite choice on FA transport into mitochondria: progenitor-like vCMs overexpressed acetyl-CoA carboxylase (ACC, *ACACA*, and *ACACB*) (inhibit) while differentiated vCMs expressed its counterparts, malonyl-*CoA* decarboxylase (MCD, *MLYCD*) (promote). Upregulation of MCD was reported to be accompanied with the enhancement of the oxidation of FAs [57]. This may heavily influence the flux of ATP synthesis, rendering as different mitochondria energetic status. These features may contribute to the low basal metabolic rate and increased size of the giant panda heart (see the “Discussion” section). In addition, as to the re-esterification of fatty acyl CoA and export, two vCMs both expressed genes encoding apolipoprotein (apo) family, MTTP, LCAT, and CPT-1, showing a relative capacity on TG and acyl CoA clearance (Fig. 3I).

### Nutrient delivery network mediated by ECs

**Fig. 4:**
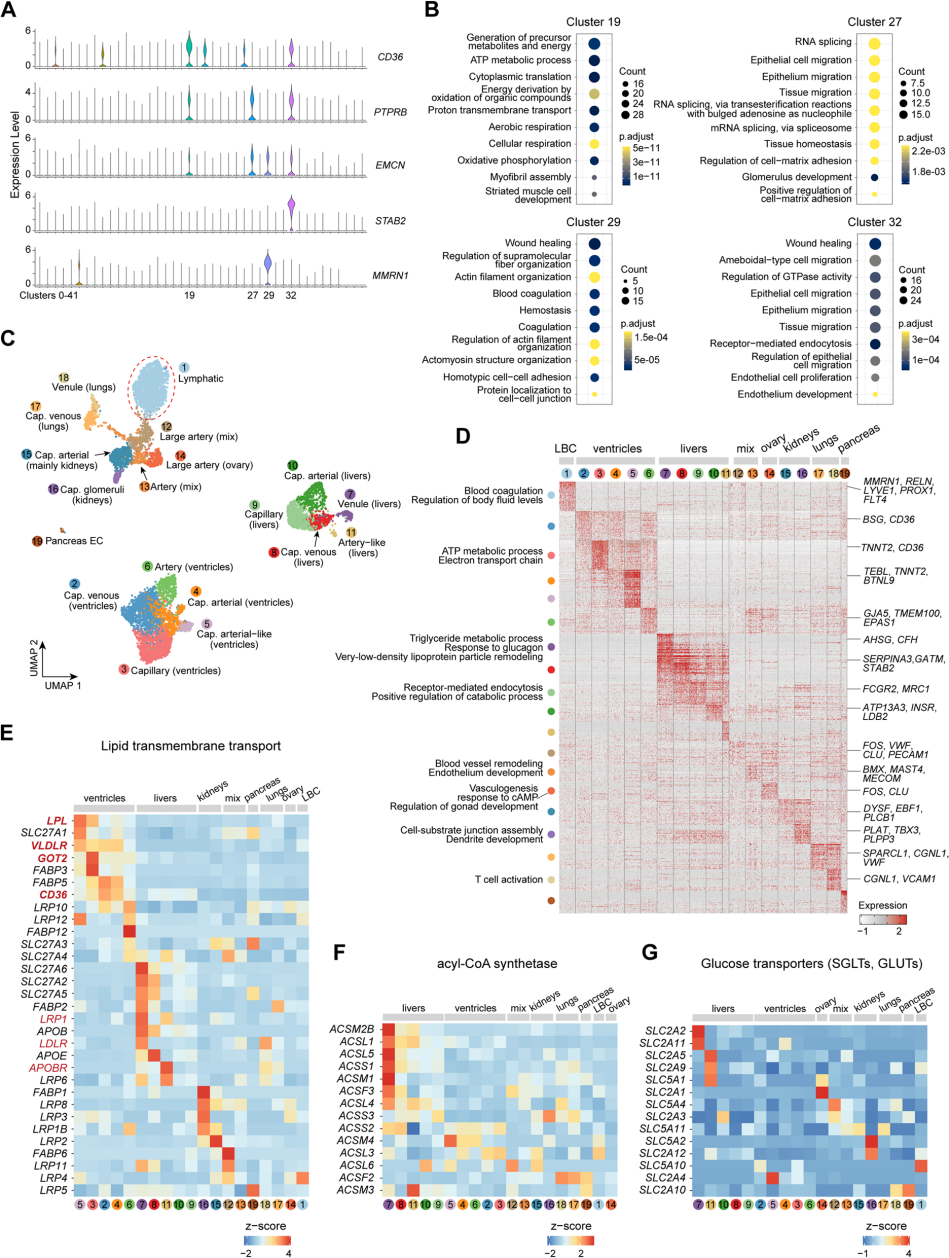
Global EC transcriptomic atlas. **A** Violin plots of canonical markers to select EC clusters from the global atlas. **B** Function diversity for EC clusters from different tissues, cluster 19 from ventricles, cluster 29 from the uterus, cluster 32 from livers, and cluster 27 represented mixed tissues. **C** UMAP visualization of EC subtypes from four large clusters in (**A**). **D** Heatmap showing the expression of the top 50 DEGs of all EC subtypes (subsample: 300) in (**C**) with representative GO terms and marker genes on each side. **E**–**G** Expression level of genes involved in lipoprotein uptake (**E**), FA activation (**F**), and glucose transport (**G**)

In adult tissues, most blood endothelial cells (ECs) are at their quiescent state, but metabolically active. Previous single-cell transcriptome studies have revealed the important roles of metabolic adaptation and plasticity of ECs in healthy and diseased organs [58–60]. Reasoning that the giant panda’s ECs play essential roles in their metabolic adaptation, we next explored the metabolic heterogeneity in their ECs. Four clusters were identified as ECs from the global sn-RNA atlas based on the expression of canonical EC markers (*PECAM1*, *VWF*, *CD36*, *PTPRB*, *FLT1*, *EMCN*, *STAB2*) (Fig. 4A). Clusters 19, 29 and 32 were predominantly contained tissue-specific ECs (ventricles, uterus, and livers, respectively) while cluster 27 represented a mix of remainder tissues, which was also observed in *Tabula Muris* [61] and the murine EC atlas [19]. Functional analysis of DEGs (Fig. 4B) revealed that mixed ECs highly expressed gene sets involving tissue homeostasis, endothelium development, actin filament-based transport, and vasculogenesis, which may represent similar functions of large vessel ECs [19]. Ventricles ECs highly expressed gene sets related to ATP metabolic process, oxidative phosphorylation, myofibril assembly, and muscle contraction, showing an important role in mitochondrial metabolism and vascular transport. Uterus ECs highly expressed gene sets involved in wounding healing, blood coagulation, platelet aggregation, and response to steroid hormone. Livers ECs highly expressed genes involved in receptor-mediated endocytosis, the establishment of endothelial barrier, response to insulin, positive regulation of the catabolic process, and intestinal lipid absorption, in agreement with liver’s functions on nutrient substance metabolism and interaction with the gastrointestinal system [16].

Endothelial cells are highly heterogeneous depending on the vascular bed or physiological conditions. We re-clustered all the ECs (summing up to 8548 nuclei) and unraveled the variation in cell heterogeneity from 20 tissues (except duodenum) (Additional file 1: Fig. S11A). Referring to established markers in mouse [19], we identified 19 EC subtypes consisting of traditional phenotypes from different vascular beds (arteries, capillaries, veins and lymphatics) (Fig. 4C). Lymphatic ECs (LECs) were distinct from blood vascular ECs (BECs), which had a more general role of regulated body fluid levels [62]. Here, LECs reached the highest proportion in the uterus (93.10%), followed by the right lung (15.28%), ovary (7.60%), esophagus (7.14%), left lung (3.31%), bladder (3.28%), left kidney (2.52%), tongue (2.50%), left ventricle (0.33%), and left liver (0.13%) (Additional file 1: Fig. S11B), suggesting a different EC subclass organization of uterus. For BECs, two populations representing large arteries and arteries were mixed ECs from various tissues (Additional file 1: Fig. S11A); it was reasonable when considering the relatively similar transcriptome profiling of arterial ECs [19]. However, large artery ECs of the ovary clustered as a distinct population with markers related to response to cAMP and regulation of gonad development (Fig. 4C, D). We also obtained capillary glomeruli, which putatively affected the dendrite development of cells in its microenvironments (i.e., PODs). BECs of ventricles and livers showed a relative distance from other tissues and predominant expression of the top 50 marker genes. Their capillary and vein subtypes showed detailed regulatory functions for the whole organ, for example, capillary and capillary arterial had shared roles in receptor-mediated endocytosis and positive regulation of the catabolic process, which may be responsible for immunity and dietary digestion of the liver [16].

Previous sc-RNA research comprehensively revealed that BECs were tightly related to specialized physiological metabolism in tissue microenvironments, and demonstrated that metabolic genes contributed largely to the tissue-grouping phenomenon of them [19]. In our dataset, heart and liver BECs showed an elevated expression for the trafficking of lipids but exhibited various transmembrane transport for targeted lipoproteins (Fig. 4E). Cardiac capillary subtypes expressed high levels of signatures for hydrolysis (*LPL*) and higher efficiency (*VLDLR*, *GOT2*, and *CD36*) for TGRLP, which was the major source of fatty acid to cardiomyocyte from the circulation [63]. Venous and artery-like ECs of livers highly expressed low-density lipoprotein receptor (*LDLR*) and apoB/E receptor (*APOBR* and *APOER*, also named as *LRP1*), which was for dietary triglyceride (TG)-rich lipoproteins. Additionally, they showed a remarkable role in fatty acid activation by acyl-CoA synthetase (Fig. 4F). Then, capillary glomeruli showed features for lipoprotein and glucose transporters (Fig. 4E, G), in line with the glomerular filtration in kidneys. We also observed that the GLUT1 (*SLC2A1*) and SGLT1 (*SLC5A1*), very important for glucose transport into the brain and uterus [64, 65], were both highly expressed in the large artery of the ovary, indicating a nutrient-absorbing capacity of the gonad, also observed in mouse testis (*Slc2a1*) [19].

### The capacity for resources exchange of guts

Lastly, we examined the expression of genes on canonical sweet and bitter signaling pathways in the gastrointestinal tract. However, few nuclei expressed *TAS1R2* and *TAS1R3* while the TAS2R gene family, *TAS2R41*, *TAS2R39*, *TAS2R4*, *TAS2R1*, and *TAS2R40* were expressed in enterocytes from the colon, endothelial cell from the duodenum, SMC from the tongue, etc. in different frequencies and levels. Genes on the downstream taste sensing pathway, such as *ITPR1*, *ITPR2*, *ITPR3*, and *TRPM5*, were expressed in some nuclei (Additional file 1: Fig. S12A). Considering the sweet taste receptor-independent pathway, we screened those genes encoding glucose transporters (GLUTs) and sodium-glucose-linked transporters (SGLTs) (Additional file 1: Fig. S12B). They were remarkedly expressed in cells from digestive organs. In particular, *SLC5A4* had the highest expression, mainly enriched in all cell types from the duodenum and enterocyte from the colon. All these above-mentioned gene expression patterns may form a broad cellular basis for gut taste-sensing functions.

## Discussion

We developed a comprehensive sn-RNA atlas of 21 commonly studied organs across seven systems and finally presented a global landscape of 185,186 nuclei for the giant panda. Our dataset was valuable and powerful for understanding giant panda physiology at single nucleus resolution, which contributed to the interspecies adaptive analysis and represented a landmark study in cellular transcriptomic signatures of wildlife.

By conducting a cross-species comparison of several tissues, our dataset exhibited the feasibility to integrate different species, which was also demonstrated by recent researches [46, 66]. This was an important step to illuminate the divergence of cellular type and expression for distinct physiological and biological traits. Substantial specialization of cellular function in organs may be attributed by multifaceted changes, including new cell subtypes, the abundance of conserved cell types, variation in cell heterogeneity, and reorganization of molecular features [66]. Here we do not pay much attention to new subtypes or cellular fractions among different datasets, because the former did not necessarily implicate species-specificity [66] and the latter was heavily influenced by the dissociation method and capture platform [67]. Some cell types with irregular dendrite were hard to dissociate out through scRNA-seq technologies while showing well outcomes in snRNA-seq (i.e., POD: 2.24% in our work and 2.4% in snRNA mouse atlas [68], versus ~0.18% in scRNA atlas [69]). Our sn-RNA dataset offered obvious advantages to investigate these special cells that we are interested in, including hepatocyte, POD, and vCM, allowing precise dissection of the cellular metabolic signatures. Therefore, we focused on the species-specific expression within certain cell types serving for essential biological life activities, which might represent different cell states of homologous cells and be associated with different lifestyles.

Low basal metabolic rate was beneficial for organism’s survival under nutrient shortage, discovered in some wild species, i.e., the folivorous red panda (*Ailurus fulgens*), the frugivorous binturong (*Arctictis binturong*) and the three-toed sloth (*Bradypus tridactylus*) [70]. Here, as one of the most talked-about issues of the giant panda, we explored several organs and discovered that the expression pattern of hepatocytes in the liver might be directly connected with food nutritional level. Hepatocytes exhibited comparable transcriptomic divergence in crab-eating monkeys and giant pandas. In giant pandas, catabolism was more significant to stimulate ATP production, together with other adaptive metabolism exemplified by amino-acid betaine and toxin metabolic processes. Low metabolism-related processes, particularly autophagy, lysosome biogenesis, and lipolysis, were promoted in giant pandas, featured by the switch on of AMPK. Additionally, related transcription factor EB (*TFEB*) were overexpressed, which appeared partially independent of mTORC1 [71]. Subsequent increased levels of *CARM1*, coactivated with *PPARG*, could promote adipocyte differentiation. Nevertheless, our study demonstrated notable molecular differences in the liver, plausibly answering the species specificity to a moderate extent.

A progenitor-like population of vCMs was identified in both the left and right ventricles of the giant panda, which may be responsible for the increased size of the giant panda’s heart. On one hand, this subtype specifically expressed *FOXO3*, *FOXO1*, and other genes related to its feedback pathways, which had roles in cardiac cell growth [56]. On the other hand, fatty acid transport into their mitochondria was repressed by ACC and the accumulation of lipids may also contribute to vCM growth. It has been proved in mouse cardiac model that ~75% of activated FAs was transported into mitochondria and 15% could be used to produce phospholipid and cholesterol ester, which were the components of the membrane [72]. Another 10% was transformed into triglyceride, which served as an energy storage material. We suspected an optimization of FA utilization in the ventricles of giant pandas by dividing it into two separate processes. Transmembrane transport and activation were accomplished in progenitor-like vCMs while free FA were transported into differentiated vCMs for mitochondrial oxidation by high expression of *GOT2*. This phenomenon may decrease the speed of FA consumption to live on a low-energy-supply diet and reduce ATP support for physical activities. And possibly, unoxidized FAs in progenitor-like vCMs could maintain mitochondrial respiration in case severe starvation might occur. Lastly, their potential for acyl CoA re-esterification and TAG transfer may be beneficial for the health and lifespan of giant pandas [15].

Resident tissues exchange nutrients, gas, and wastes with blood vessels, especially in capillaries. ECs have essential roles in maintaining microenvironmental homeostasis and substance transport. Specialized expression in BEC subtypes from livers and ventricles showed interacted fatty acid metabolic processes. Venule and capillary venous ECs in livers were important for triglyceride-rich lipoprotein particle remodeling, cholesterol efflux, sterol transport, and negative regulation of hemostasis. Capillary arterial, capillary, and capillary venous ECs overexpressed genes (*LPL*, *CD36*, *VLDLR*, and *GOT2*) responsible for TGRLP transport, recruiting lipids from blood vessels. The dysfunction of the liver-heart axis is considered to be involved in metabolic syndrome in non-alcoholic fatty liver disease, characterized by excessive hepatic accumulation of lipid [17, 18]. Our analysis of BECs provided powerful cellular clues for the normal liver-heart crosstalk to maintain lipid homeostasis. As for reproductive organs in our dataset, a comparable fraction of large artery ECs were obtained from the ovary and they highly expressed glucose transporters for nutrient uptake. But the major subclass in the uterus was LEC, providing a cellular clue on energy limit. Insufficiency of BECs may cut off the nutrient supply, leading to a low energy expenditure on the uterus in the unpregnant stage for giant pandas. But its LECs still had a role in cellular response to vascular endothelial growth factor stimulus, potentially implying the ability of angiogenesis in the pregnant season. The changes in cell organization in the uterus need further stronger evidence from younger or pregnant donors.

Sugar is the primary energy source and gut cells also could activate neurons via the gut-brain axis, mediating the sugar-preference signaling circuit [73]. For the giant panda, we demonstrated the high-level expression of genes encoding glucose transporters in cells from the duodenum and enterocytes from the colon and also expressed in gastric mucosa cells from the stomach. We supposed that these molecules were effective and helpful to sense and uptake sweet substances from the low-glucose bamboo.

Lastly, one possible application of single cell/nucleus transcriptomic atlas is to localize the cellular tropism of the virus infection based on the expression of viral receptor-encoding genes. In wild populations, viruses with high morbidity and mortality rates could take a heavy toll on animals [74]. So, we mapped the expression of 79 genes encoding receptors of 78 viruses [75] and reported the cellular susceptibility in giant pandas (Additional file 1: Fig. S13-15). With respect to previously reported infectious viruses, the left lung seemed to be the most potential organ accessible to influenza A virus, parvoviruses, and rotavirus, with cell targets being AT1, AT2, MP, and their proliferative cells, which viruses could directly reach. Rare cell types were also likely to be virus targets, such as PODs, overexpressed *ITGB1*, *ITGB5*, *DPP4*, and *NCAM1*, compared to other renal cells.

This work also faces limitations. Firstly, the detected genes per nucleus in this giant panda atlas were relatively low, therefore we avoided any hypotheses or conclusions related to under-detected features. Secondly, this atlas was not designed to investigate a single organ in detail, so cell types were not comprehensive in each organ. Thirdly, we noticed that the proportion of reads mapped confidently to intergenic regions was high, accounting for 48.02% ± 1.44% of all reads mapped confidently to the genome (Additional file 2: Table S1). Although nuclear RNA is especially rich in non-coding sequences, with 41% consisting of intergenic sequences and 25% intronic sequences [76, 77], we cannot exclude the possibility that some genic regions were not successfully predicted in the reference genome. Fourthly, it is important to declare that cellular fraction might be biased for some hard-to-dissociate cell types and tissues using snRNA-seq/scRNA-seq, including the kidney, heart, liver, and brain. For cells that are large (muscle cell), fragile (neuron, adipocyte), tight with adjacent cells (POD, hepatocyte), and multinucleated (muscle, trophoblast), snRNA-seq is more powerful than scRNA-seq while scRNA-seq can recover more immune cells [67, 68, 78]. Buffers and wash conditions are essential to obtain high-quality and purified nuclei and cells, which need to be modified for different tissues. Fluorescence-activated cell sorting (FACS) is an effective method to remove large fibrosis and debris, especially in the heart tissue isolation process [29]. Finally, animal tissues only came from a 31-year-old female donor after an hour of natural death. Further work should be conducted on more individuals of different ages and genders using combined snRNA-seq and scRNA-seq technologies with optimized dissociation protocols and additional functional validation.

## Conclusions

Our study broadened and deepened the cellular knowledge of fitness optimization in the giant panda by dissecting essential metabolic cell types and processes. We uncovered the low metabolic signatures in the liver, fatty acid oxidation process in the ventricles, and nutrient adsorption and delivery networks by gut and ECs. This resource would undoubtedly enhance the understanding of the unique characteristics of non-model species, providing insights for further studies on ecological adaption and evolution.

## Methods

### Collection of giant panda tissues

All tissue samples were isolated from a female giant panda that died naturally at the China Conservation and Research Centre for the Giant Panda. Collected organs/tissues included the heart, lung, kidney, liver, trachea, bladder, pancreas, thyroid, ovary, uterus, stomach, duodenum, colon, spleen, esophagus, and tongue. Samples (10 g) of each organ/tissue were obtained, quickly frozen in liquid nitrogen, and stored at −80 °C until nuclear extraction.

### Preparation of nucleus suspensions

We isolated nuclei according to the protocol described by Bakken et al. [79]. Frozen tissues were placed into a homogenization buffer, minced, and homogenized using a homogenizer on an ice-cold board to release nuclei. Lysates were filtered through a 30 μm cell strainer and centrifuged for 5 min at 4 °C. The supernatant was discarded, and nuclei were resuspended in a cell resuspension buffer. Samples were centrifuged again, and the nuclei were re-suspended at a concentration of 1000 nuclei/μL for single-nucleus library preparation.

### Single-nucleus library preparation and sequencing

Single-nucleus libraries were prepared using the DNBelab C Series Single-Cell Library Prep Set (MGI Tech, Shenzhen, China, #1000021082) [80]. Single-nucleus suspensions, functionalized beads, and lysis buffer were encapsulated into the emulsion droplets. A single nucleus was lysed, and mRNA transcripts were captured by the bead in each droplet. Transcripts were linked to the sequencing adaptor, cell barcode, UMI, and oligo-dT. Then, the emulsion was broken, beads were collected, and reverse transcription was performed to generate cDNA molecules. Afterwards, cDNA amplification, enrichment, and purification were conducted before sequencing libraries were constructed. Single-nucleus RNA libraries were prepared according to the manufacturer’s instructions. Libraries were sequenced on a DIPSEQ T1 sequencer (BGI, Shenzhen, China).

### Generation of a single-cell matrix from raw sequencing data

Considering that our tissues were processed by snRNA-seq, we created a “pre-mRNA” reference concluding both exons and introns using the mkref function by CellRanger software. Raw sequencing reads were aligned to the Ame_Sichuan reference genome [81, 82] and converted to expression matrix using a modified Cell Ranger count pipeline based on the STAR software (version 2.7.4a) [83].

### Doublet removal and quality control

Firstly, doublets were detected and removed for each library respectively using DoubletFinder in R (version 4.0.2) [84]. The threshold was set automatically based on the predicted detectable doublet fraction. Then, we performed standard quality control based on gene number and the ratio of mitochondrial genes in each nucleus using the Seurat package (version 4.0.2) [85] in R. Nuclei with more than 200 detected genes and percent_mito < 5% were maintained.

### Unsupervised clustering and cell type annotation

Seurat package was applied to perform unsupervised clustering. Counts were log-normalized for each nucleus, and 2000 highly variable genes were selected using the FindVariableGenes function. For batch effect correction, we used the FindIntegrationAnchors function to identify anchors across sequencing libraries of the same tissue and integrated them using the IntegrateData function. Then, the scaleData function was employed to regress RNA counts. Principal component analysis was performed on the scaled data, and ElbowPlot was used to assess the significance of principal components. The cell clusters were identified using the FindClusters function with the resolution set to 0.5 and visualized using t-Distributed Stochastic Neighbor Embedding (t-SNE) and Uniform Manifold Approximation and Projection (UMAP). Finally, marker genes of each cluster were identified using a Wilcoxon rank-sum test implemented in the FindAllMarkers function with the parameters: only.pos = T, min.pct = 0.25, logfc.threshold = 0.25. Cell clusters were annotated and referred to canonical markers of specific cell types from an extensive literature search (Supplementary Table 3). For the global atlas, we also used the above workflow to integrate all tissues and perform clustering and annotation while the resolution was set to 1 in the FindClusters process. To compare the annotation results between single and global atlas, we calculated the ratio of each cell type from a single organ atlas in the global clusters and visualized them by heatmap.

### Cross-species comparison

Interspecies analysis was performed among human (HCL), mouse (MCA), monkey (NHPCA), and giant panda. Our dataset was preprocessed following the same steps in a previous study [46], in brief, only orthologous genes in the giant panda genome with the other three species and expressed in all four datasets were kept and gene names of giant panda were transformed as the same format with their previous integration work [46]. After that, the giant panda atlas was integrated with the already prepared atlas of human, mouse, and monkey, using the above-integrated pipeline in the “Unsupervised clustering and cell type annotation” section. For the biological questions we were concerned, only four organs (stomach, livers, kidneys, and uterus) were used to conduct interspecies integration. Then, cell clustering and annotation were performed and cell identity was assured based on the marker genes consistent with that in the previous three species comparison. For the prominent divergence in the liver atlas, we made a recluster analysis within the same cell type to observe conserved and specific transcriptomic features of giant pandas.

### Differentially expressed genes (DEGs) analysis

DEGs of each cluster were selected from its marker gene list with p_val_adj ≤ 0.05 and avg_logFC ≥ 0.5 [86]. GO enrichment analysis was performed on the DEGs using the package “clusterProfiler” [87, 88] in R software. Each significantly enriched category included at least two genes, and the hypergeometric test was used to estimate significance (*p* < 0.05). Networks of GO terms were visualized by REVIGO to summarize redundant terms [89].

### Pseudotime trajectory analysis

The Monocle2 package (version 2.24.1) [90] in R was applied to construct single-cell trajectories of the target cell type from a young state to a mature state. The DDRTree algorithm was used for dimensionality reduction and the trajectory was visualized using the plot_cell_trajectory function colored by “Pseudotime” and “seurat_clusters.”

### Screening the cellular target of viruses

All receptors of 78 viruses were obtained from a previous computational analysis [75] and a literature investigation in the human lung [31]. We showed the fraction and average expression level in each cell type for every virus.

### Supplementary Information

**Additional file 1:** **Fig. S1.** *t*-SNE visualization of global clustering indicating the distribution of each tissue. **Fig. S2.** Quality control for each organ/tissue. **Fig. S3.** UMAP visualization of cell clusters in each single organ atlas colored by cell identity. **Fig. S4.** UMAP visualization of unsupervised clusters and violin plots for marker genes indicating cell types. **Fig. S5.** UMAP visualization of paired tissues and their cell profiling. **Fig. S6.** Heatmap showing the expression level of marker genes from a global landscape. **Fig. S7.** Heatmap used to compare the cell annotation between single organ/tissue and the global unsupervised clustering. **Fig.** **S8.** Cross-species comparison of human, mouse, monkey and giant panda for stomach, liver, kidney and uterus. **Fig. S9.** UMAP visualization of human, mouse and monkey cells/nuclei with previously published cell annotation results. **Fig. S10.** Cell types that enriched CYP family and* PAH* gene. **Fig. S11.** Unsupervised clustering of ECs colored by clusters and organ/tissue, respectively. **Fig. S12.** Expression levels of genes in taste signaling pathways and glucose transporters in gastrointestinal tract. **Fig. S13.** Possible cell targets for eight viruses infectious to giant panda. **Fig. S14.** Detection the receptors of SARS-CoV-2 virus for giant panda. **Fig. S15.** Detection the receptors of other 69 potential viruses for giant panda.

**Additional file 2:** **Table S1.** Sequencing information and output of Cellranger for each library. **Table S2.** Quality control and cell annotation results of each organ/tissue.** Table S3.** Information of canonical markers from published literature. **Table S4.** GO analysis for uncertain cell populations.

## Data Availability

The data that support the findings of this study have been deposited into the CNGB Sequence Archive (CNSA) [91] of the China National GeneBank DataBase (CNGBdb) [92] with accession number CNP0002076 [93].

## Abbreviations

AMPK: AMP-activated protein kinase
TORC1: Target-of-rapamycin complex 1
TORC2: Target-of-rapamycin complex 2
CM: Cardiomyocyte
FA: Fatty acid
FAO: Fatty acid oxidation
TGRLP: Triglyceride-rich lipoprotein
NEFA: Non-esterified fatty acid
EC: Endothelial cell
LPL: Lipoprotein lipase
scRNA-seq: Single-cell RNA sequencing
snRNA-seq: Single-nucleus RNA sequencing
UMI: Unique molecular identifier
FB: Fibroblast
MP: Macrophage
SMC: Smooth muscle cell
PTC: Proximal tubule cell
DTC: Distal tubule cell
LOH: Loop of Henle
POD: Podocyte
CD-IC-B: Collecting duct intercalated cell - type B
CD-tran-PC: Intercalated cell transiting to principal cell
HSC: Hepatic stellate cell
AT1: Alveolar epithelial type 1
AT2: Alveolar epithelial type 2
PMC: Pit mucosa cell
GO: Gene ontology
DEG: Differentially expressed gene
vCM: Ventricular cardiomyocyte
PI3K: Phosphatidylinositol 3-kinase
NFAT1: Nuclear factor of activated T cell 1
FABP: Fatty acid-binding protein
FATP: Fatty acid transporter
VLDLR: Very low-density lipoprotein receptor
FABPpm: Plasma membrane-associated fatty acid-binding protein
ACC: Acetyl-CoA carboxylase
MCD: Malonyl-CoA decarboxylase
LEC: Lymphatic endothelial cell
BEC: Blood vascular endothelial cell
LDLR: Low-density lipoprotein receptor
APOBR: apoB receptor
APOER: apoE receptor
TG: Triglyceride
GLUT: Glucose transporter
SGLT: Sodium-glucose-linked transporter
TFEB: Transcription factor EB
FACS: Fluorescence-activated cell sorting
