# Supplementary material for: Single-nucleus transcriptome inventory of giant panda reveals cellular basis for fitness optimization under low metabolism
Source: BMC Biol. 2023 Oct 20;21:222. doi: 10.1186/s12915-023-01691-2 (PMC10588165; doi:10.1186/s12915-023-01691-2)
Supplement: Supplementary file 1 — Additional file 1: Fig. S1. t-SNE visualization of global clustering indicating the distribution of each tissue. Fig. S2. Quality control for each organ/tissue. Fig. S3. UMAP visualization of cell clusters in each single organ atlas colored by cell identity. Fig. S4. UMAP visualization of unsupervised clusters and violin plots for marker genes indicating cell types. Fig. S5. UMAP visualization of paired tissues and their cell profiling. Fig. S6. Heatmap showing the expression level of marker genes from a global landscape. Fig. S7. Heatmap used to compare the cell annotation between single organ/tissue and the global unsupervised clustering. Fig. S8. Cross-species comparison of human, mouse, monkey and giant panda for stomach, liver, kidney and uterus. Fig. S9. UMAP visualization of human, mouse and monkey cells/nuclei with previously published cell annotation results. Fig. S10. Cell types that enriched CYP family and PAH gene. Fig. S11. Unsupervised clustering of ECs colored by clusters and organ/tissue, respectively. Fig. S12. Expression levels of genes in taste signaling pathways and glucose transporters in gastrointestinal tract. Fig. S13. Possible cell targets for eight viruses infectious to giant panda. Fig. S14. Detection the receptors of SARS-CoV-2 virus for giant panda. Fig. S15. Detection the receptors of other 69 potential viruses for giant panda. [file 12915_2023_1691_MOESM1_ESM.docx]

**
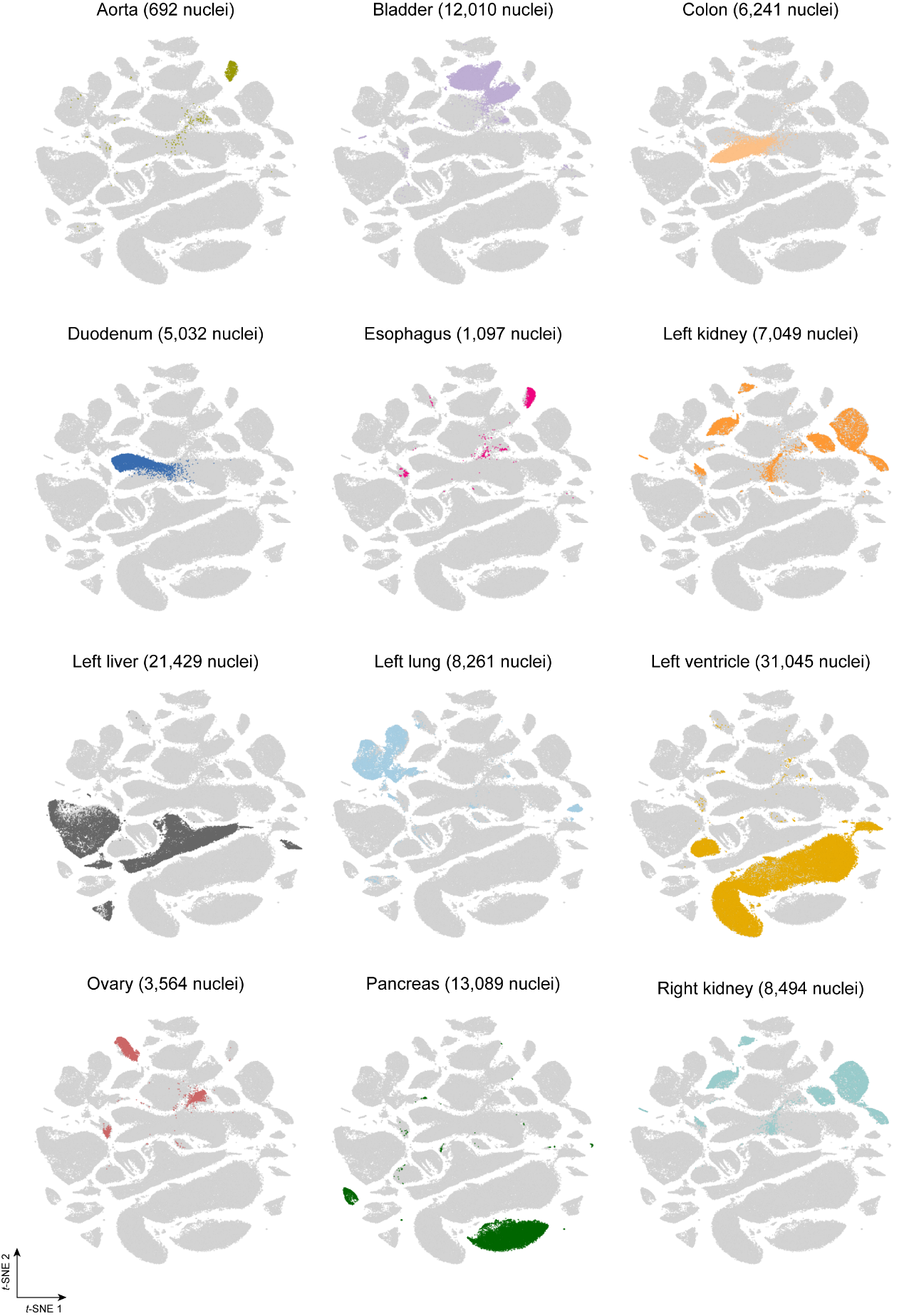
**

**
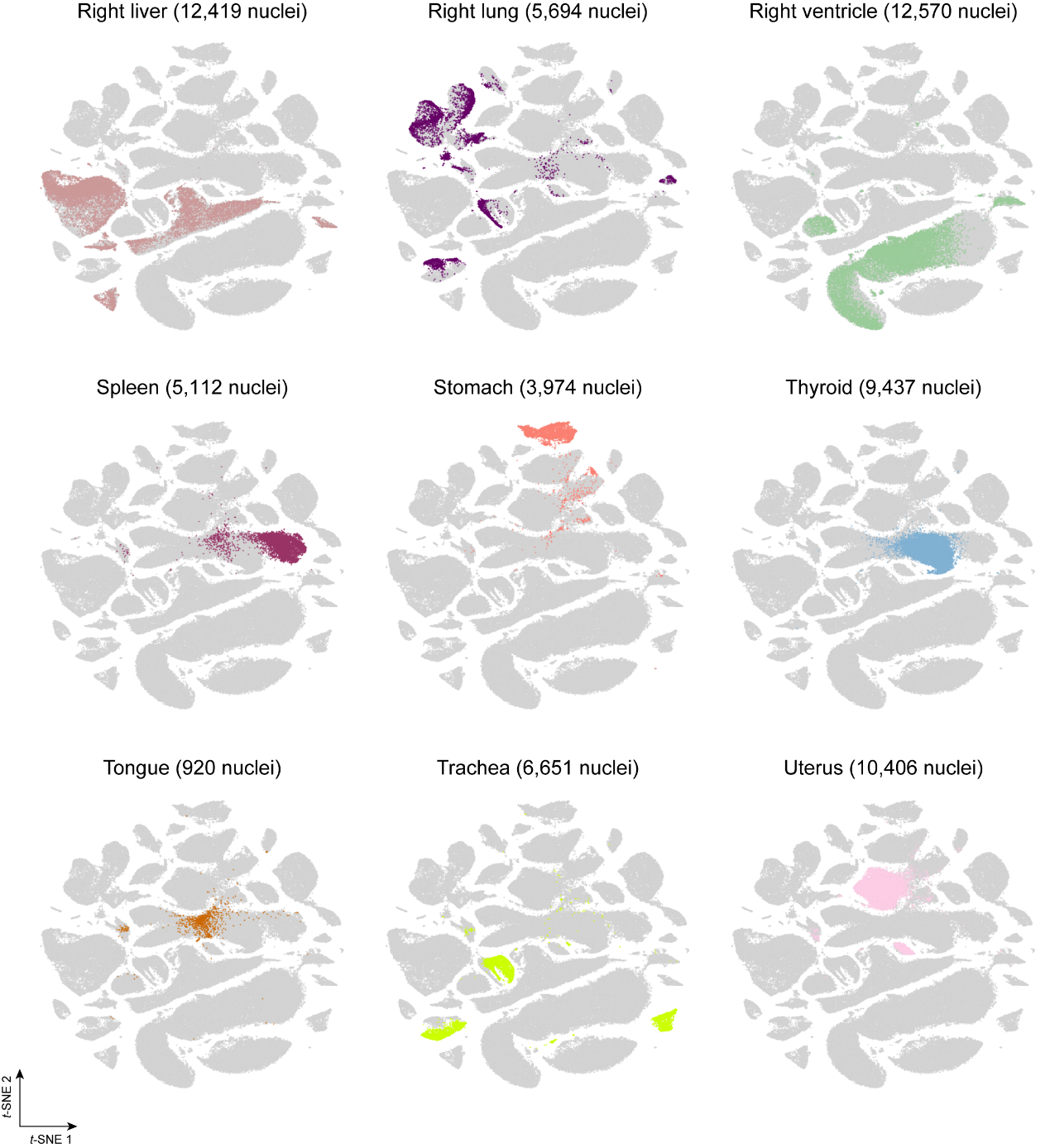
**

**Fig. S1.** *t*-SNE visualization of global clustering indicating the distribution of each tissue. Nuclei from different tissues were highlighted by distinct colors with the nuclei number on the top.

**
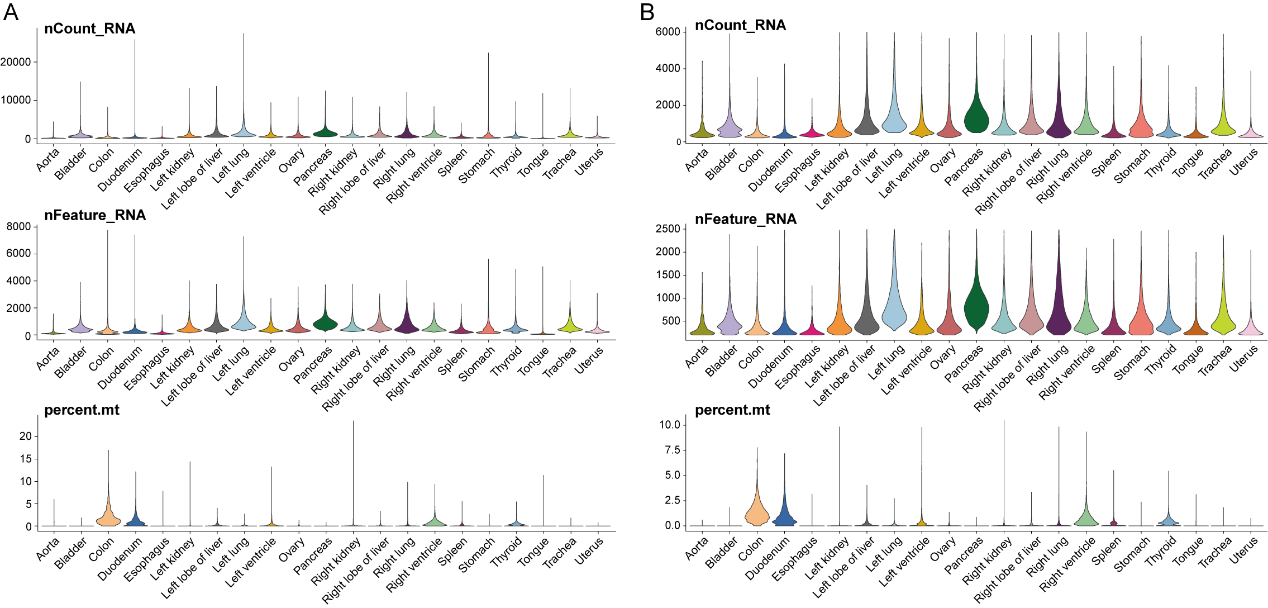
**

**Fig. S2.** Quality control for each organ/tissue. Violin plot showing the number of UMI (top), genes (middle) and ratio of mitochondrial gene (bottom) for each organ/tissue. (A) raw data from CellRanger software (B) obtained dataset after doublets removal and low-quality nuclei filtering.

**
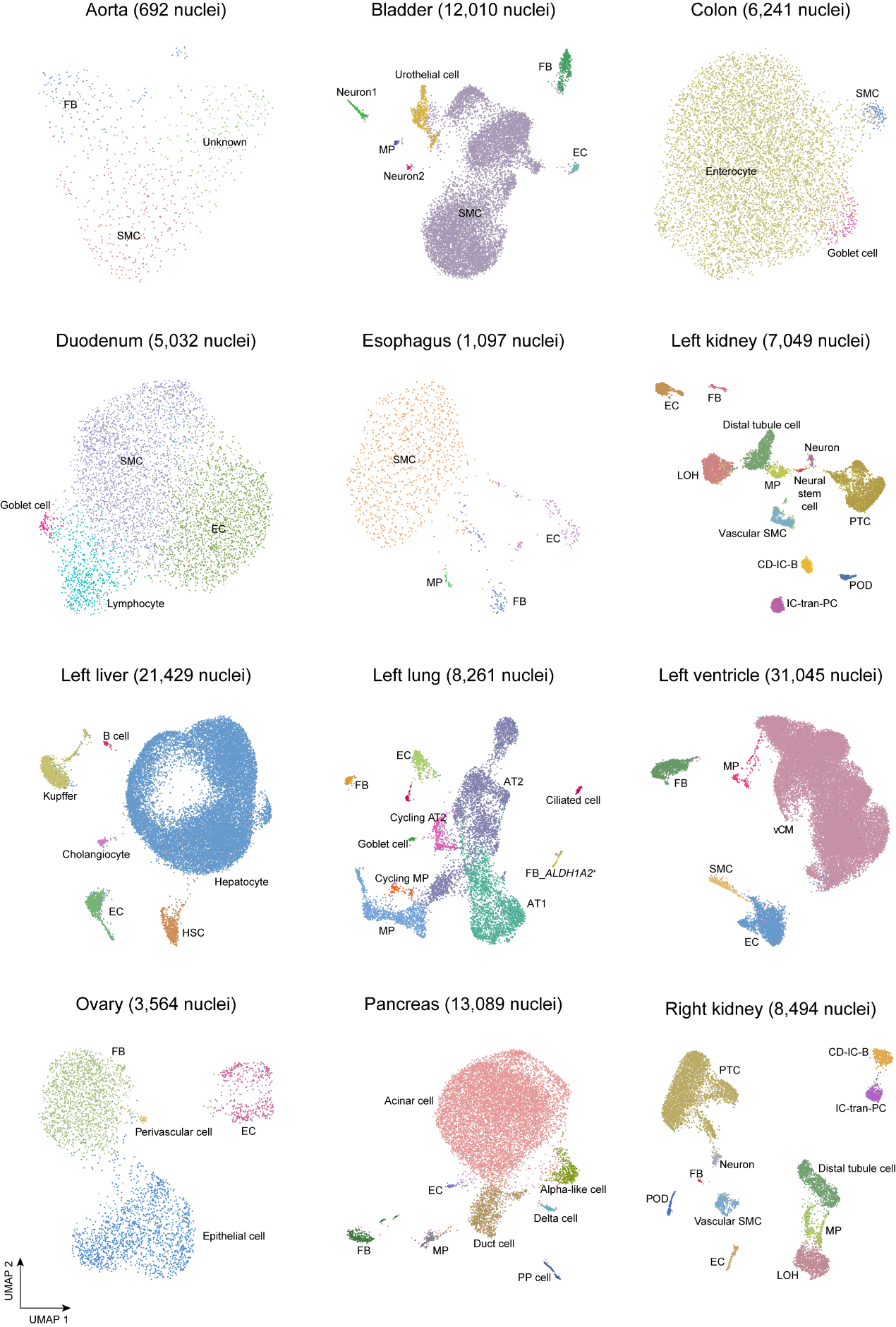
**

**
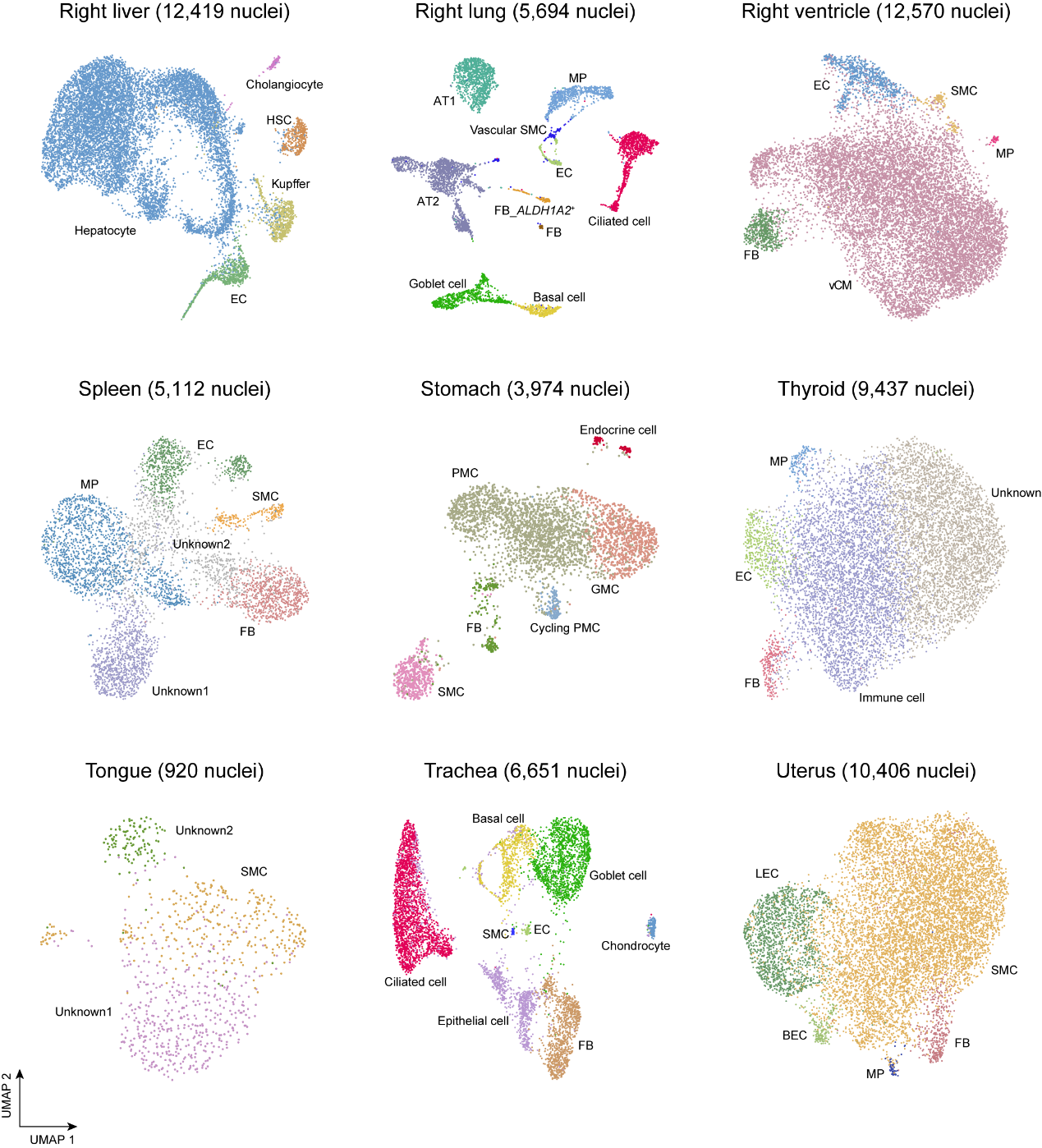
**

**Fig. S3.** UMAP visualization of cell clusters in each single organ atlas colored by cell identity.


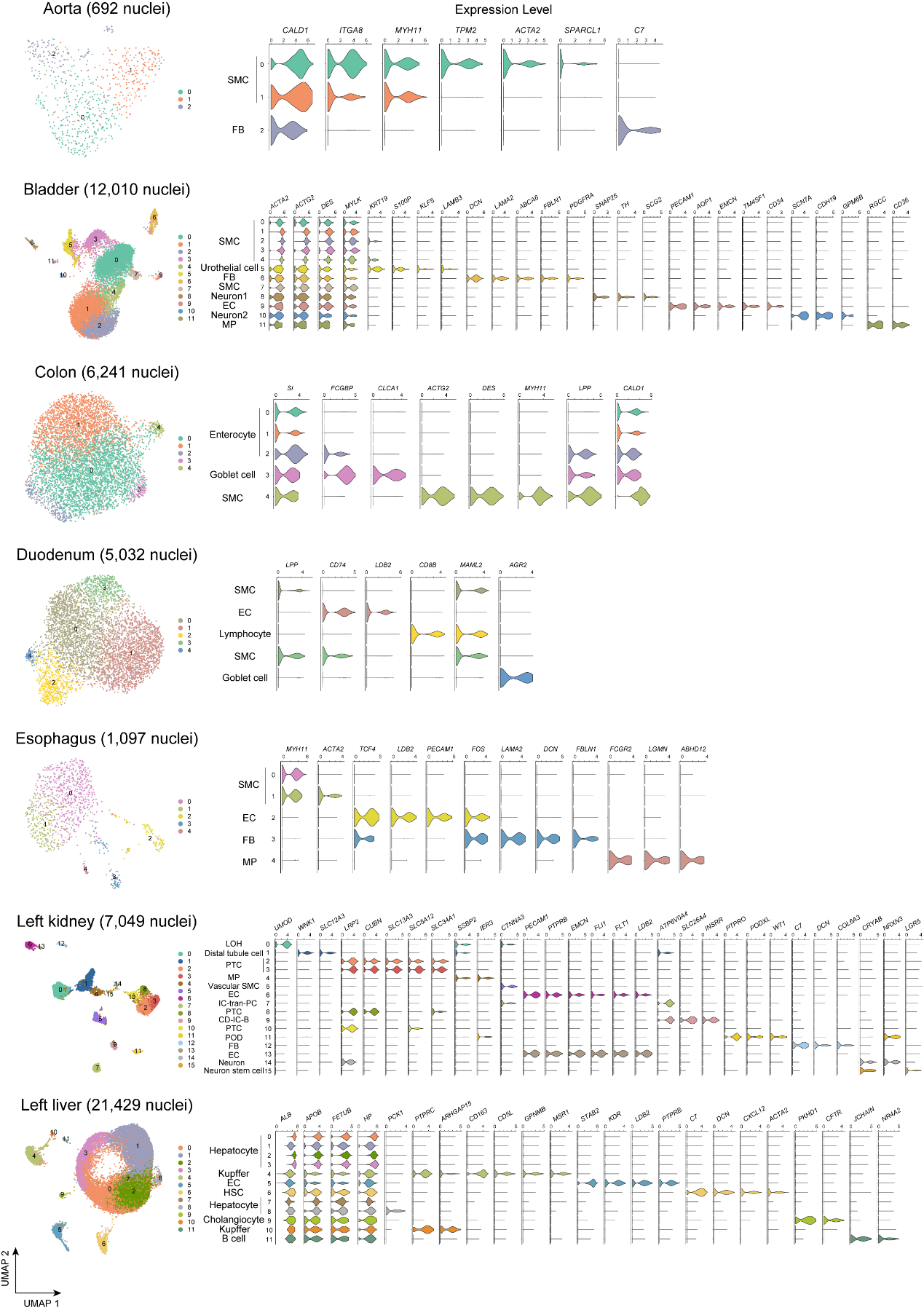


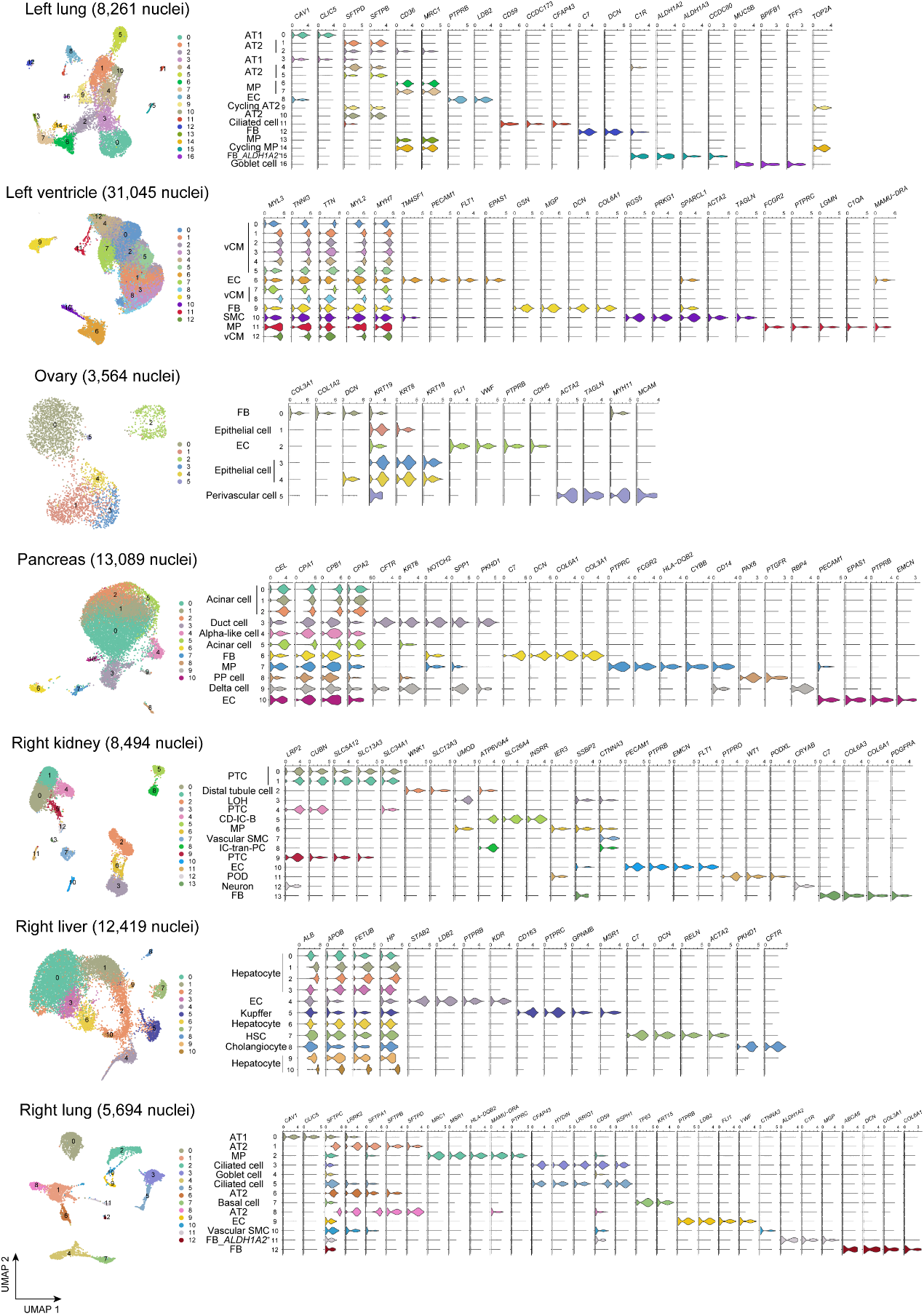


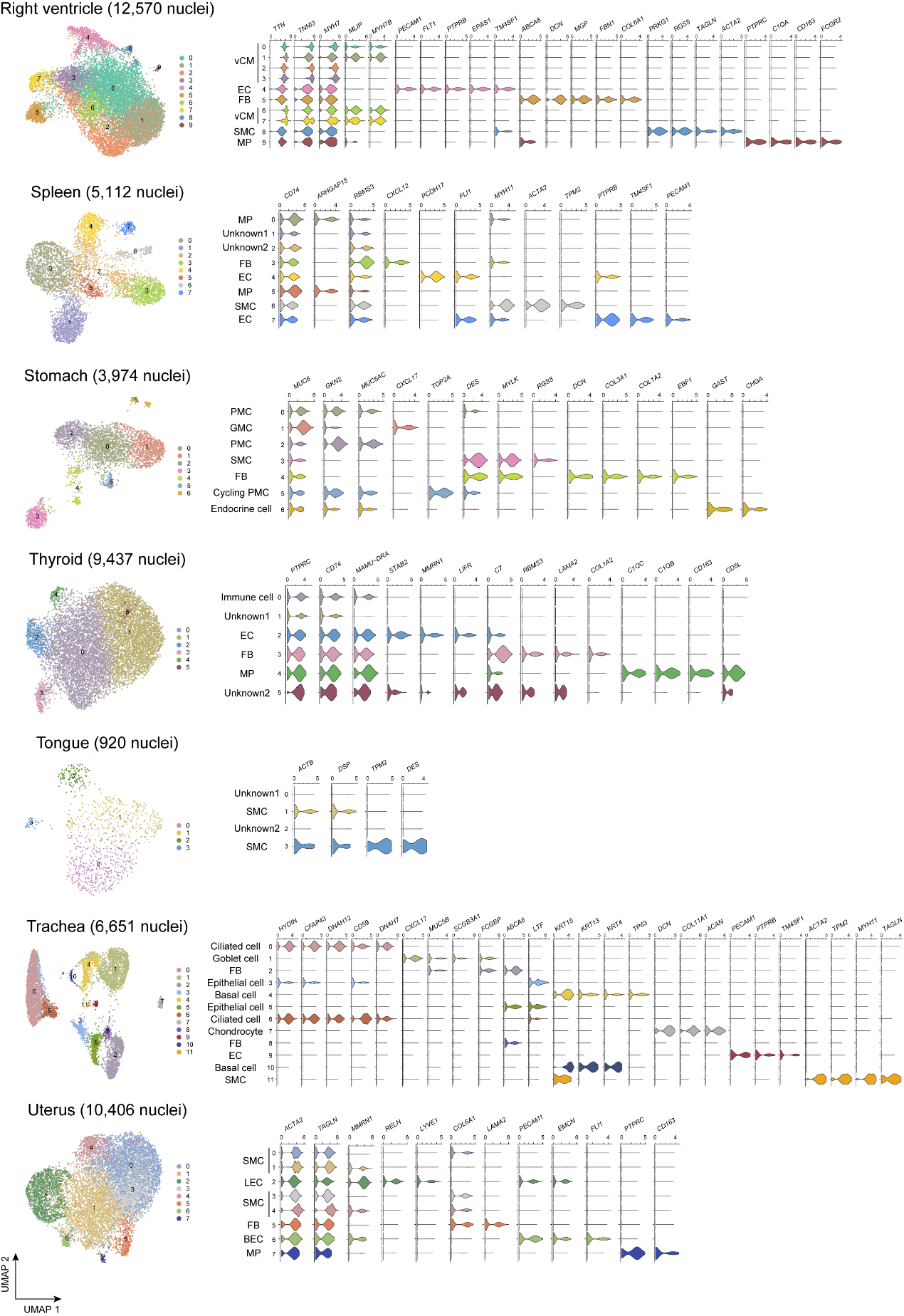


**Fig. S4.** UMAP visualization of unsupervised clusters and violin plots for marker genes indicating cell types.


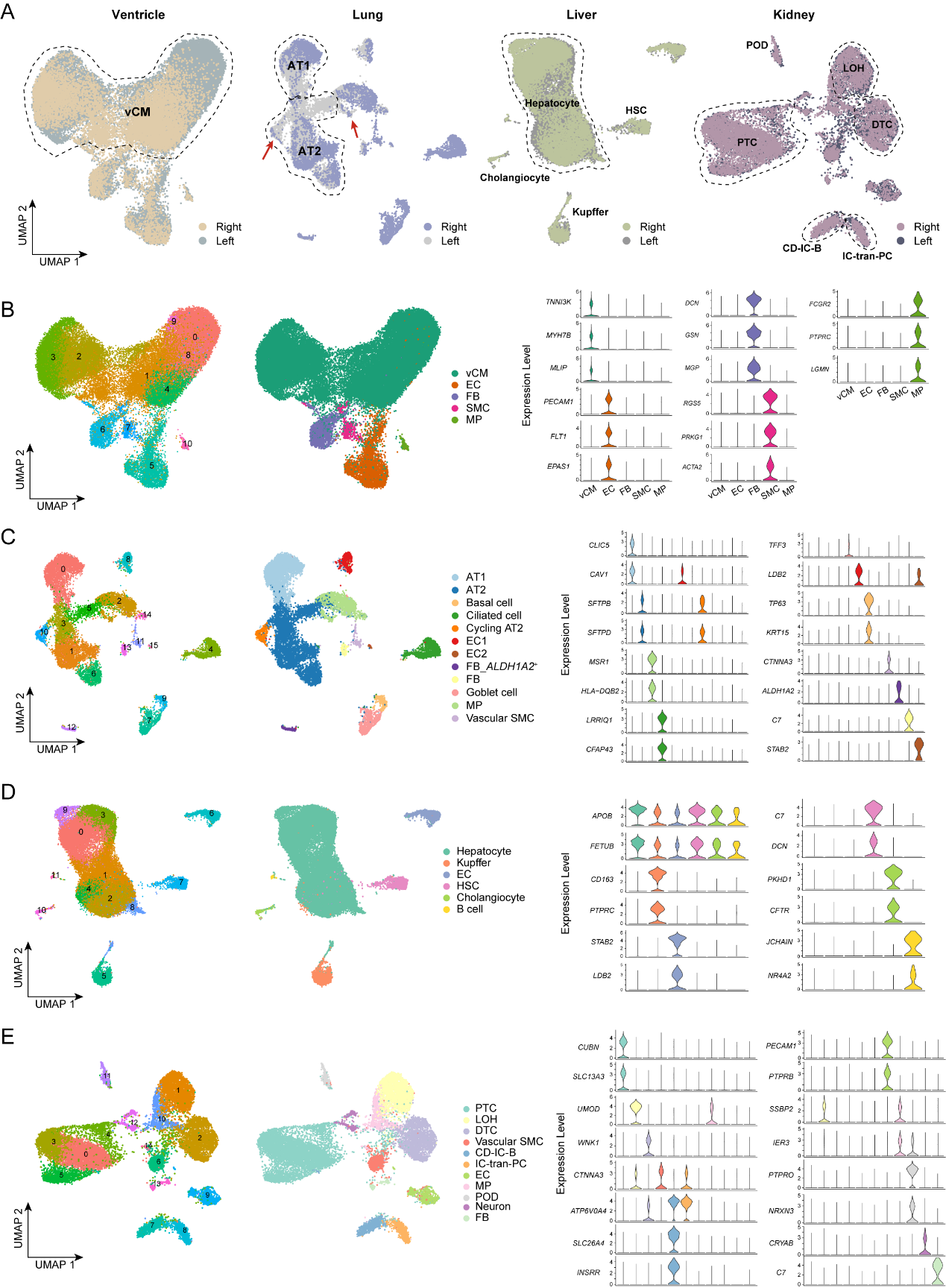


**Fig. S5.** UMAP visualization of paired tissues and their cell profiling. (A) Distribution of nuclei from left and right counterparts of paired organs. Arrows indicated cycling cells. (B-E) UMAP visualization of unsupervised clustering and cell identities for ventricles (B), lungs (C), livers (D) and kidneys (E), with violin plot of marker genes on the right.


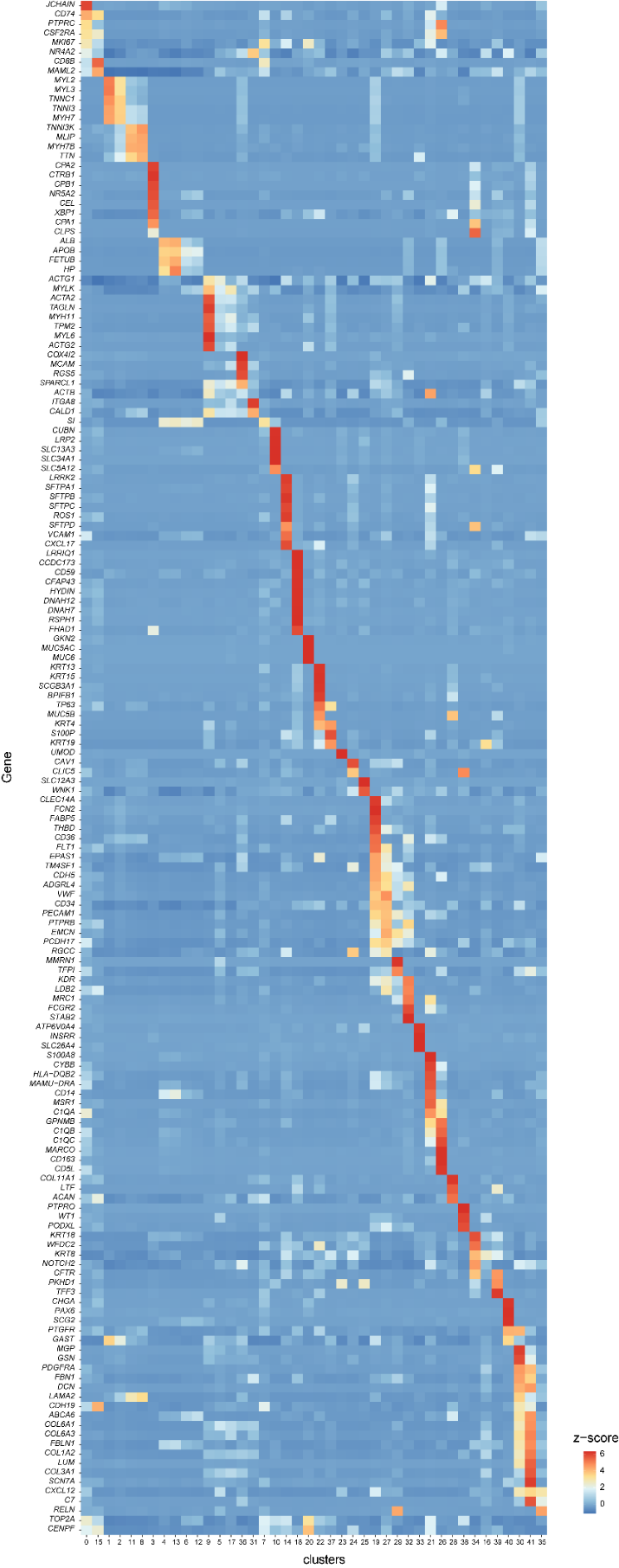


**Fig. S6.** Heatmap showing the expression level of marker genes from a global landscape.


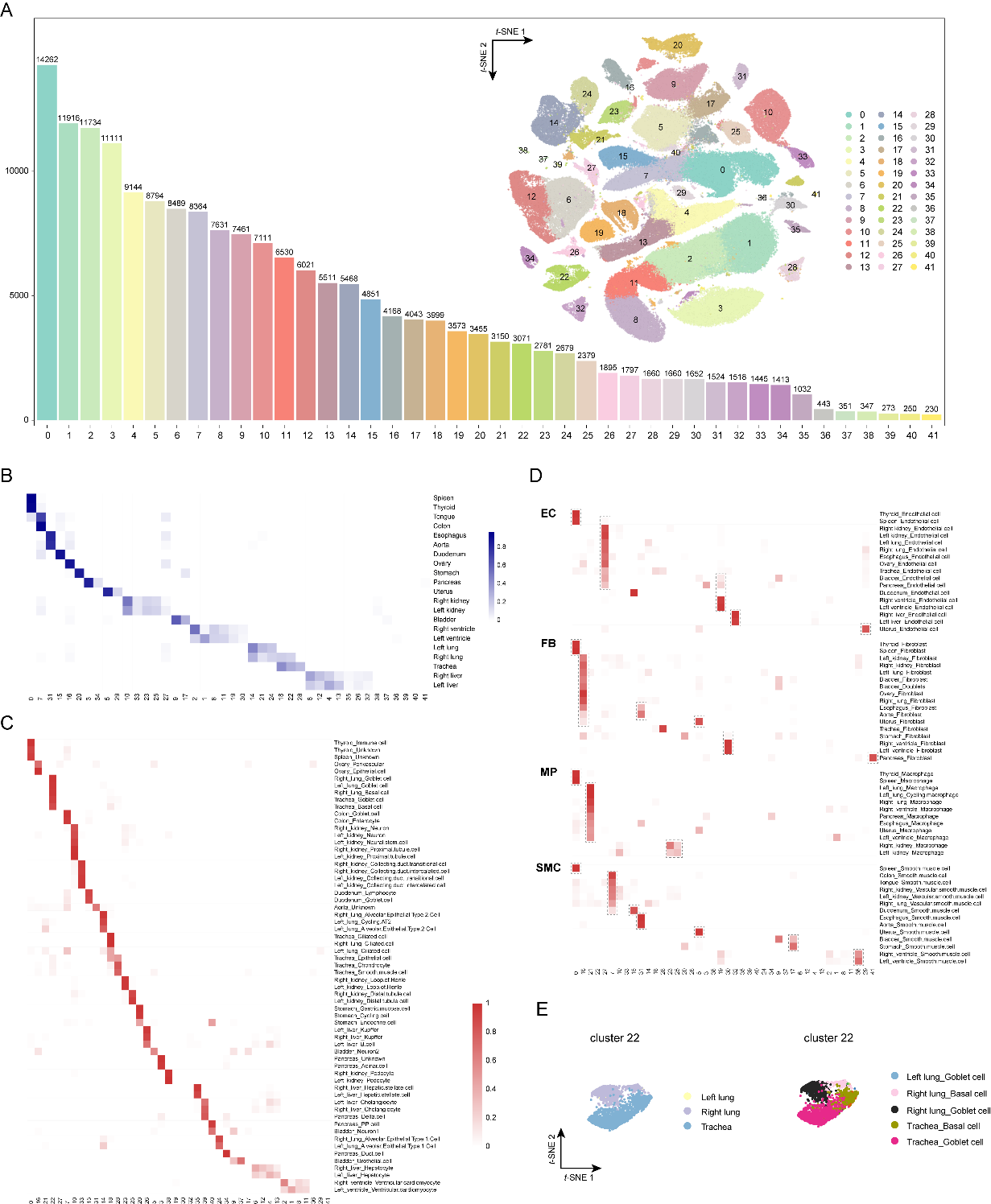


**Fig. S7.** Heatmap used to compare the cell annotation between single organ/tissue and the global unsupervised clustering. (A) Number of nuclei in each cluster of global unsupervised clustering. (B) Distribution of nuclei in the global clusters at an organ/tissue level. Blue color indicated the nuclei number of each organ/tissue that existed in each cluster, divided by the total number of the organ/tissue. (C, D) Distribution of nuclei in the global clusters at a cell type level for tissue-species (C) and common (D) cell types. Red color indicated the nuclei number of each cell type that existed in each cluster, divided by the total number of the cell type. (E) *t*-SNE visualization of the same cell types of lungs and trachea in cluster 22, colored by tissue and cell type, respectively.


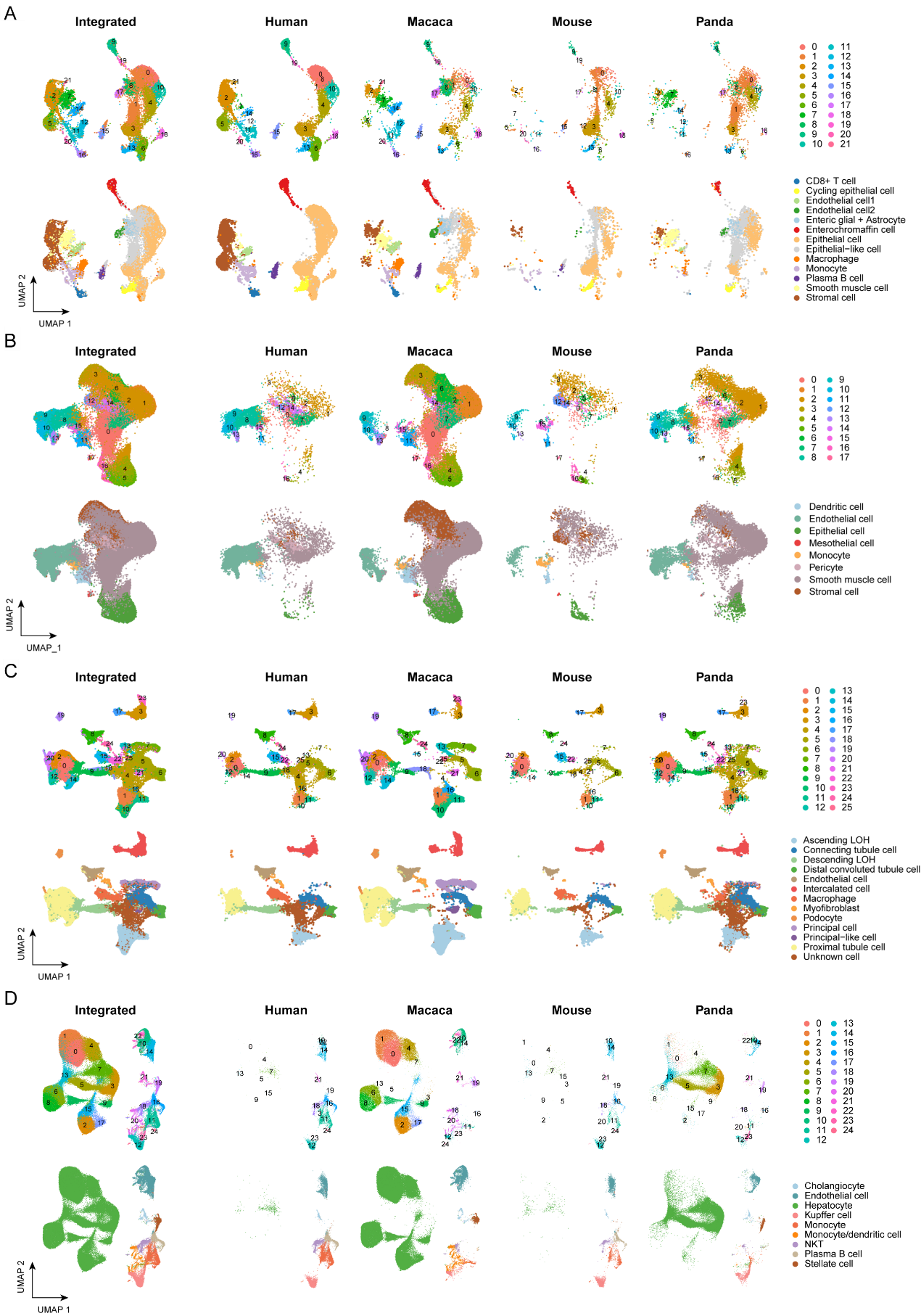


Fig. S8. Cross-species comparison of human, mouse, monkey and giant panda for stomach, liver, kidney and uterus. Unsupervised clustering and cell identity showed by integrated atlas and each species for stomach (A), uterus (B), kidney (C) and liver (D).


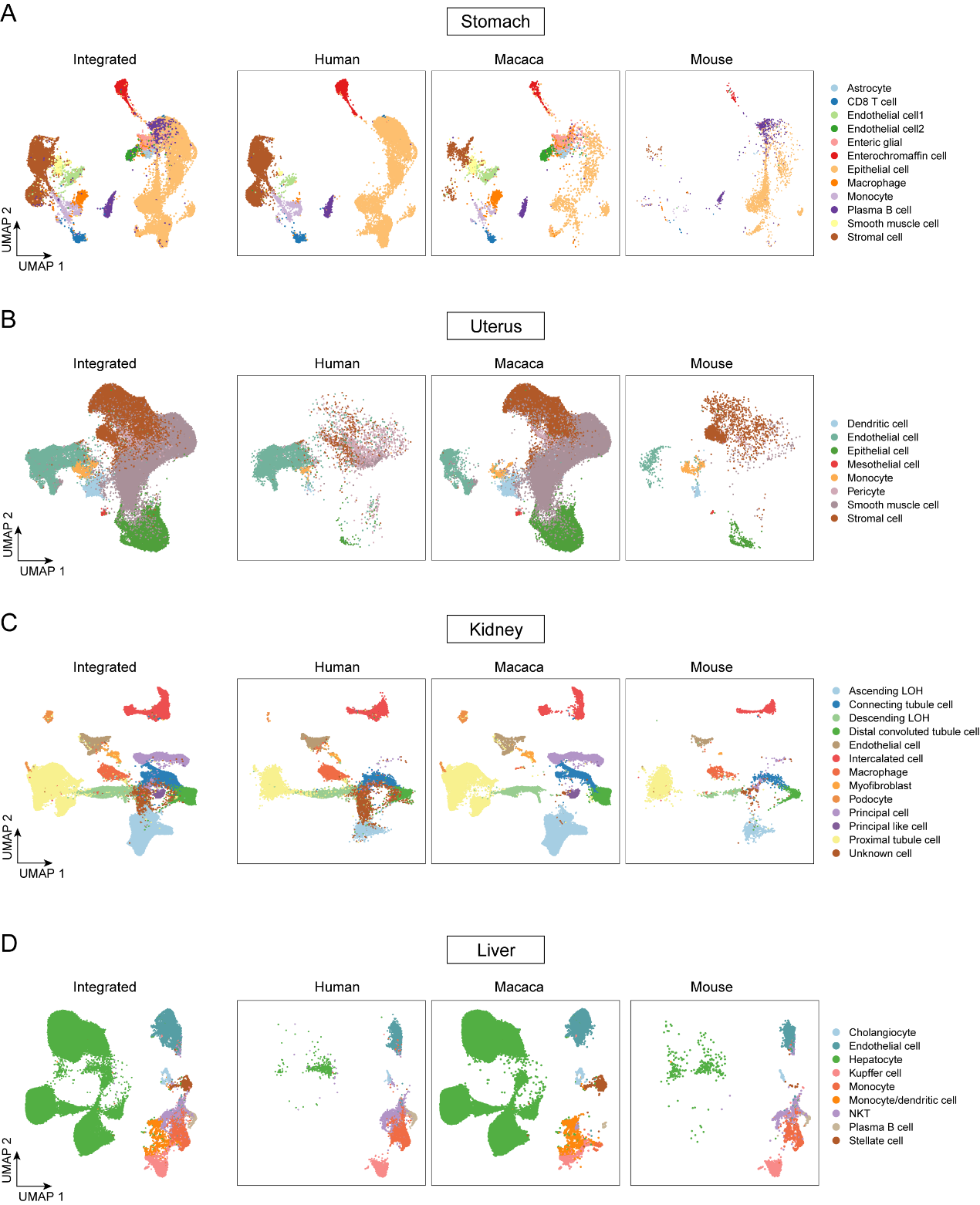


Fig. S9. UMAP visualization of human, mouse and monkey cells/nuclei with previously published cell annotation results. The clustering was based on four species’ integration while cell identity was adhered to published results in NHPCA article based on cell barcode, for stomach (A), uterus (B), kidney (C) and liver (D).


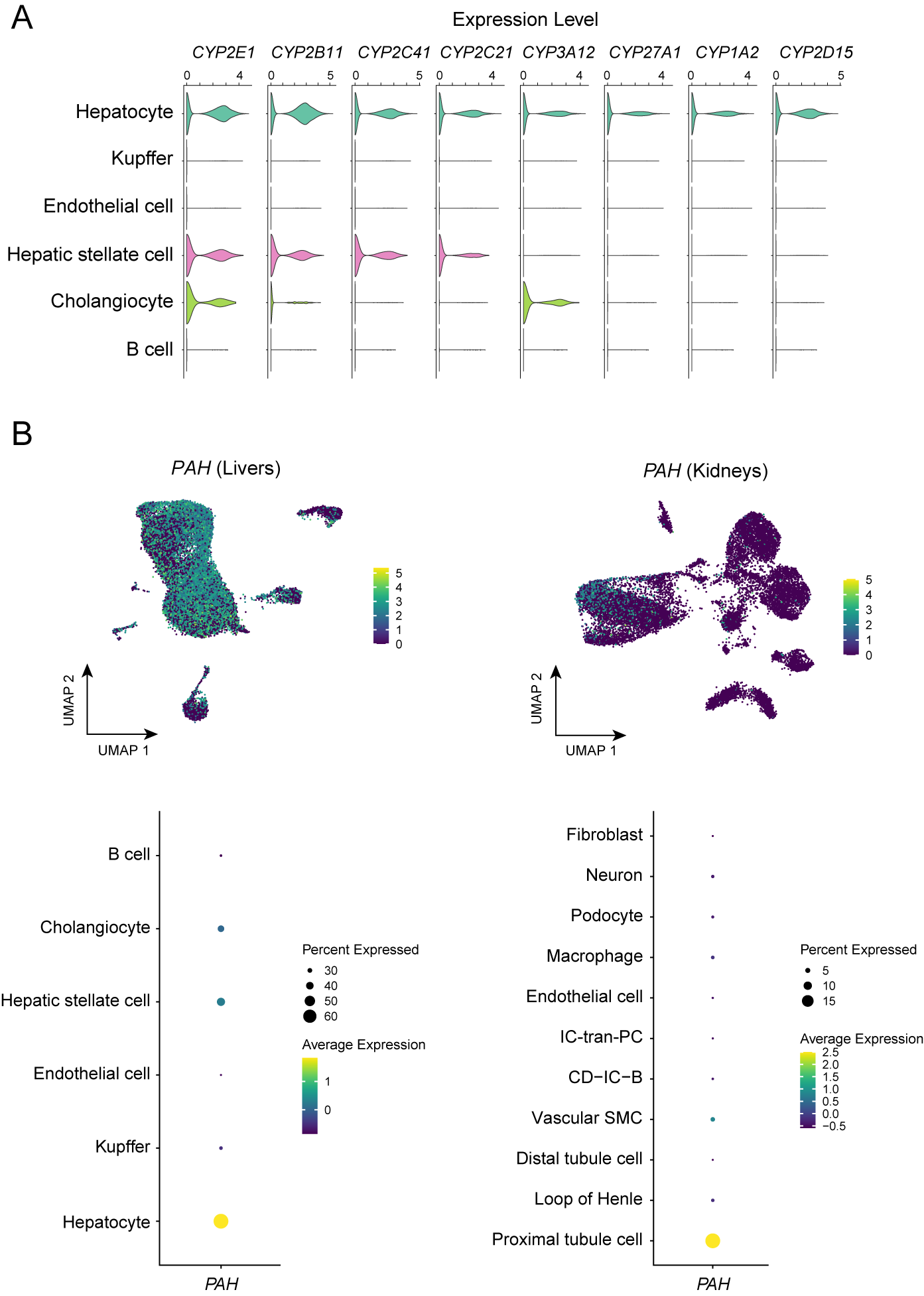


**Fig. S10.** Cell types that enriched CYP family and *PAH* gene. Violin plots showing the cell types expressed CYP family (A) and the distribution of *PAH* gene in livers and kidneys (B).


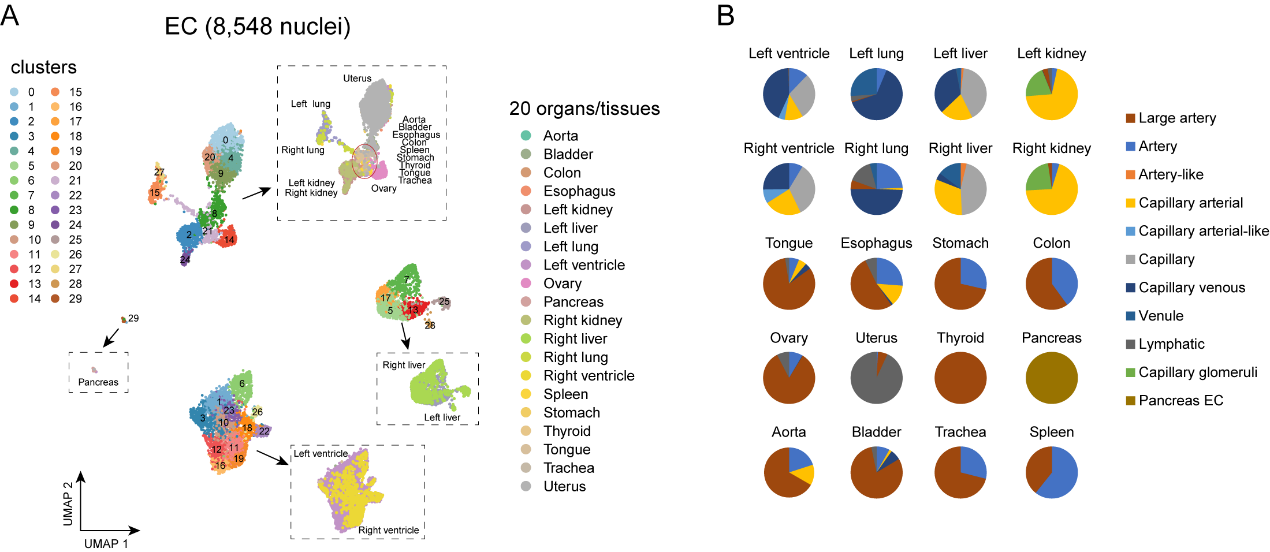


**Fig. S11.** Unsupervised clustering of ECs colored by clusters and organ/tissue, respectively. (A) UMAP visualization of re-clustering for the four EC populations from the global atlas. (B) Cell fraction of EC subtypes in each organ/tissue.


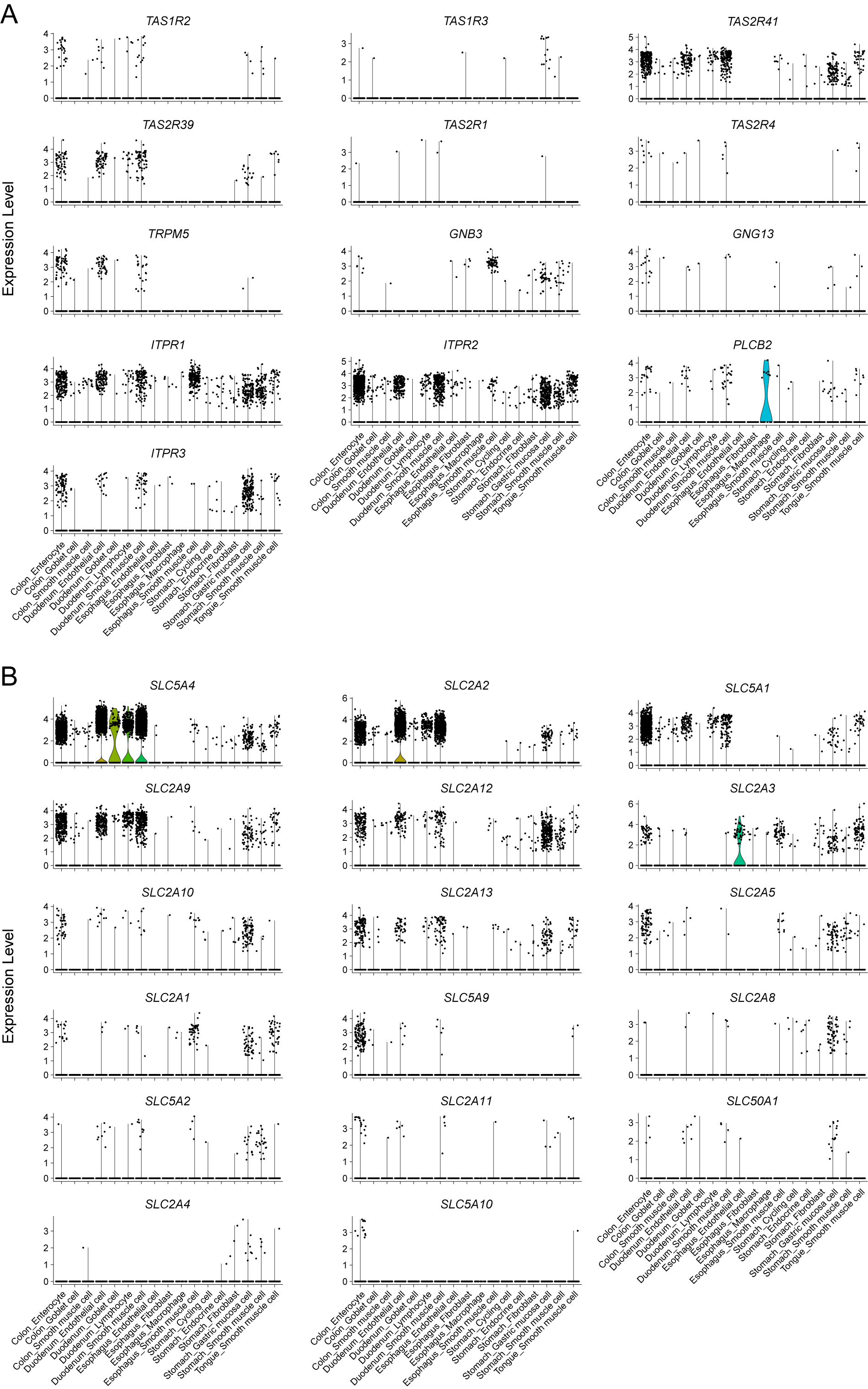


**Fig. S12.** Expression levels of genes in taste signaling pathways (A) and glucose transporters (B) in gastrointestinal tract.


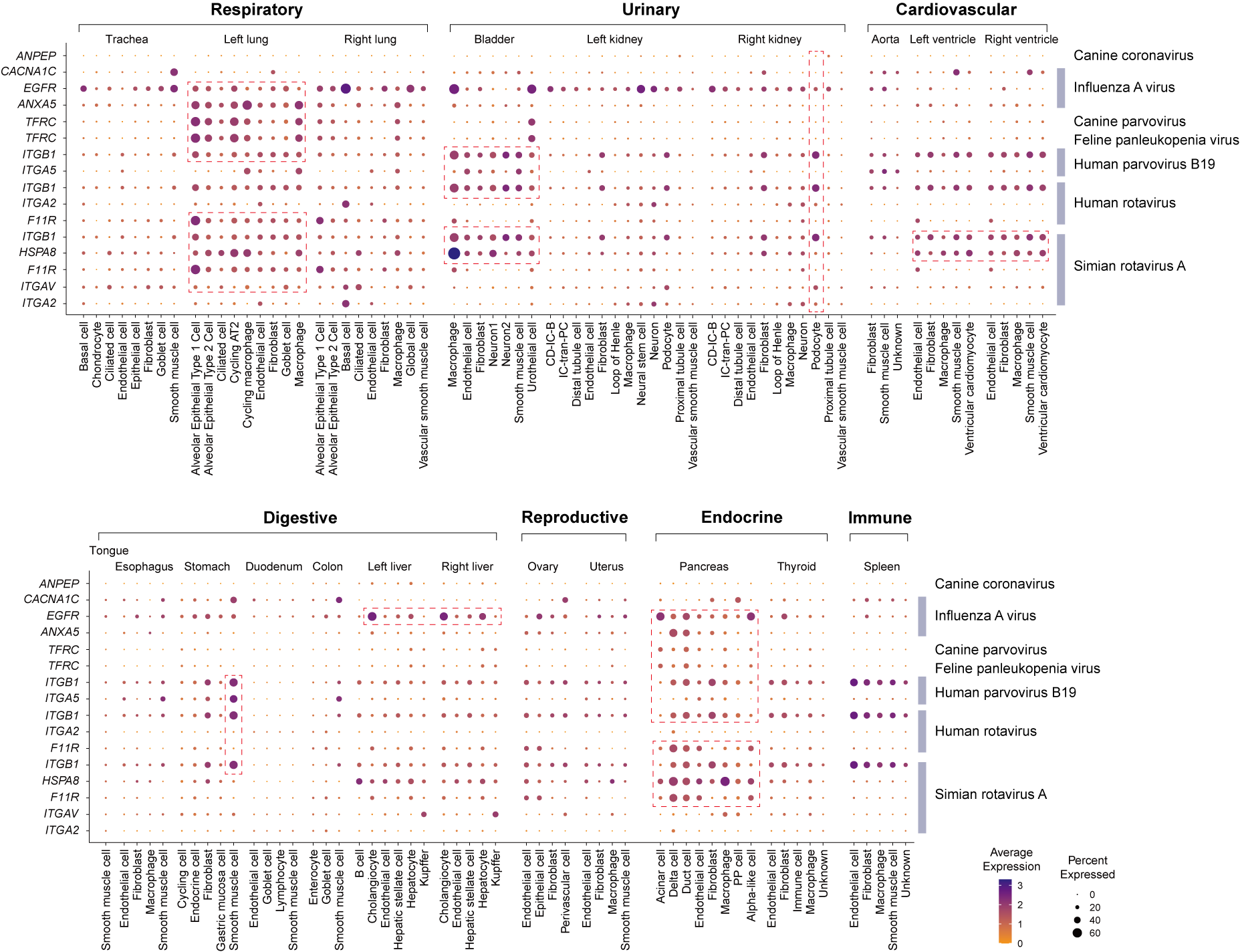


**Fig. S13.** Possible cell targets for eight viruses infectious to giant panda.


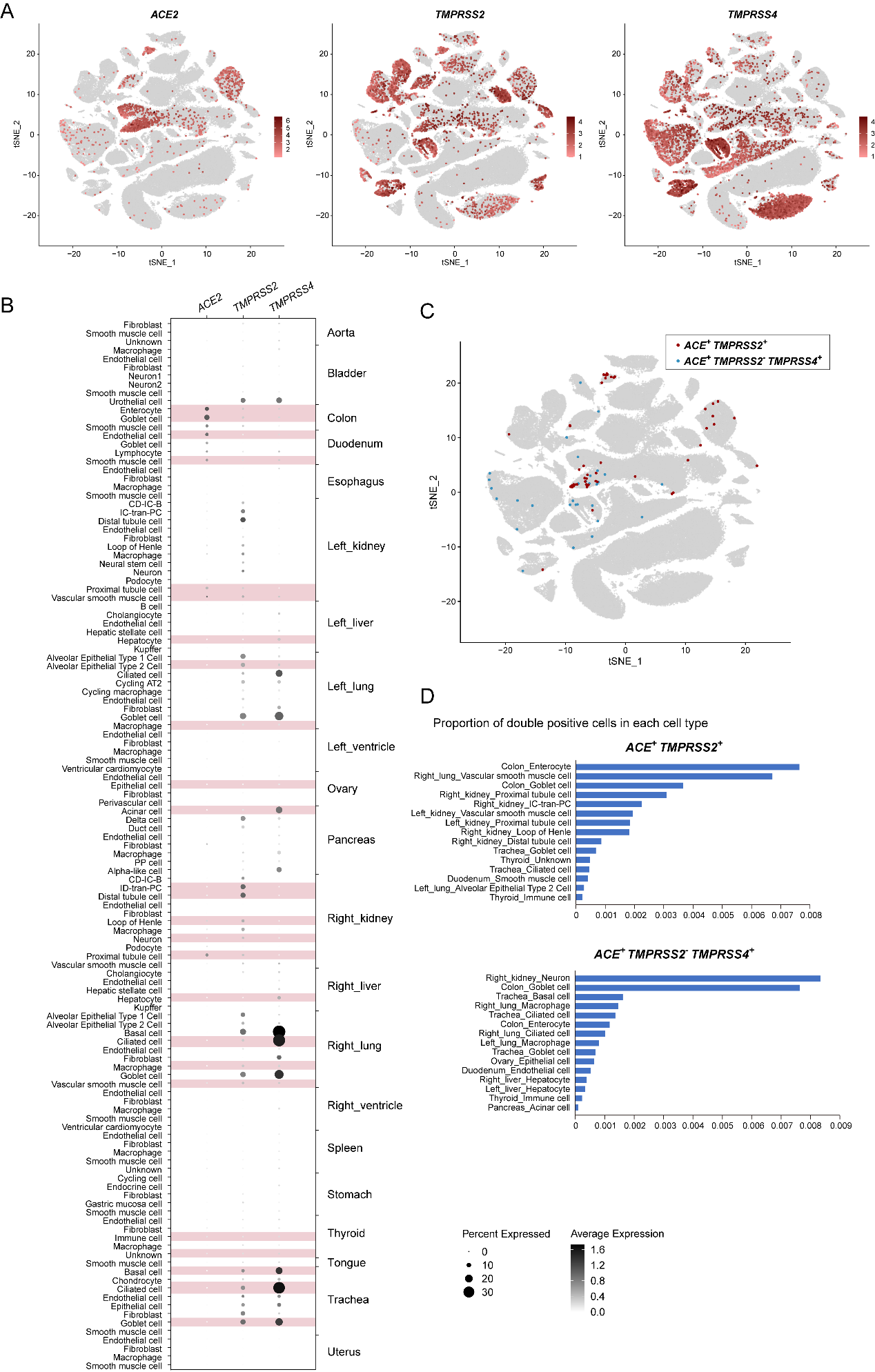


**Fig. S14.** Detection the receptors of SARS-CoV-2 virus for giant panda. (A) *t*-SNE visualization of the expression of *ACE2*, *TMPRSS2*, and *TMPRSS4* in all single nuclei. (B) Dot plot showing the expression of *ACE2*, *TMPRSS2,* and *TMPRSS4* in each cell type. Pink shading represents cell populations co-expressing *ACE2* and *TMPRSS2* or *TMPRSS4*. (C) *t*-SNE visualization of the distribution of *ACE2*^+^ *TMPRSS2*^+^ *TMPRSS4*^-^ and *ACE2*^+^ *TMPRSS2*^-^ *TMPRSS4*^+^ nuclei (colored in red and blue, respectively). (D) Bar plot indicating the proportion of *ACE2*^+^ *TMPRSS2*^+^ *TMPRSS4*^-^ and *ACE2*^+^ *TMPRSS2*^-^ *TMPRSS4*^+^ nuclei within each cell type.


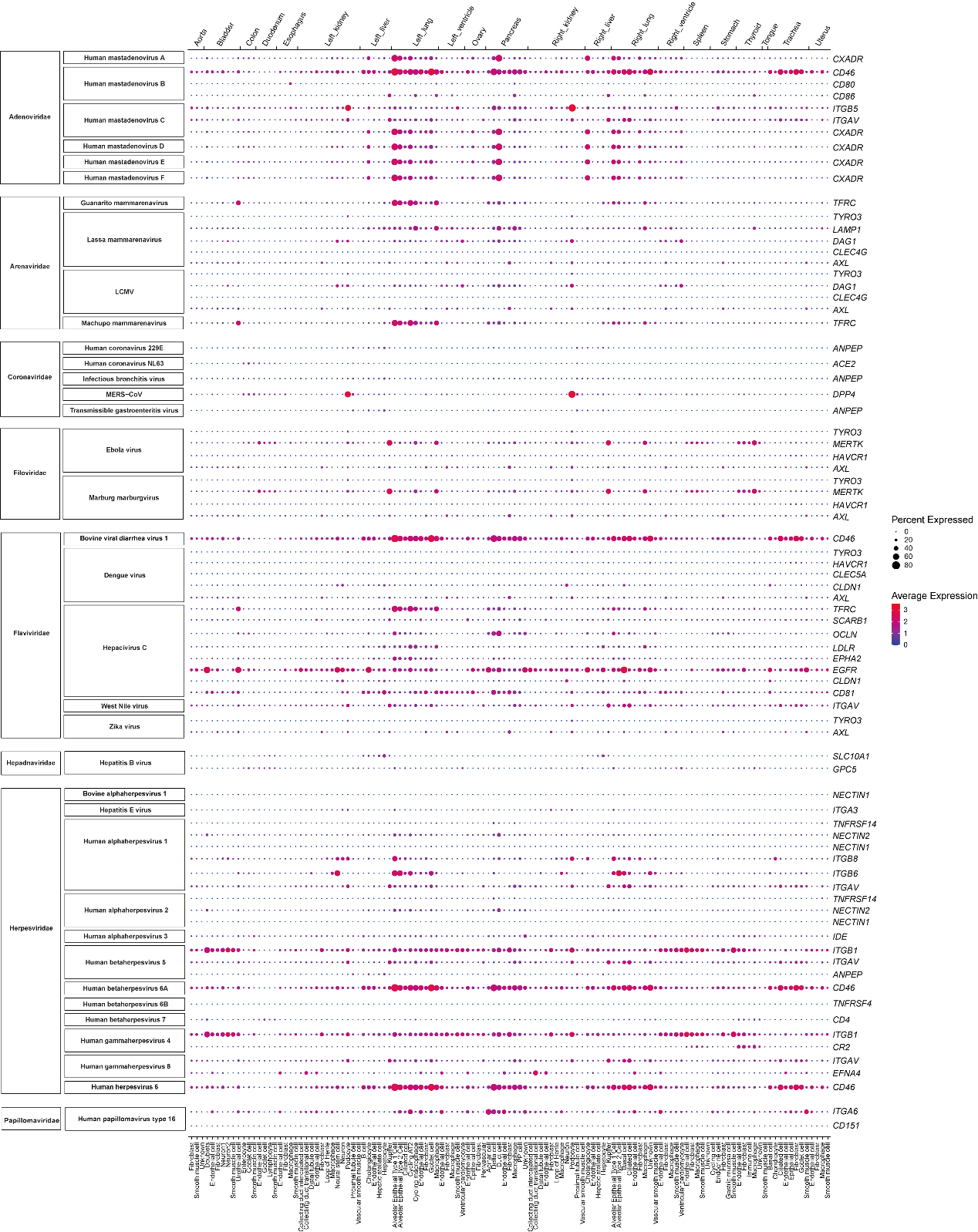


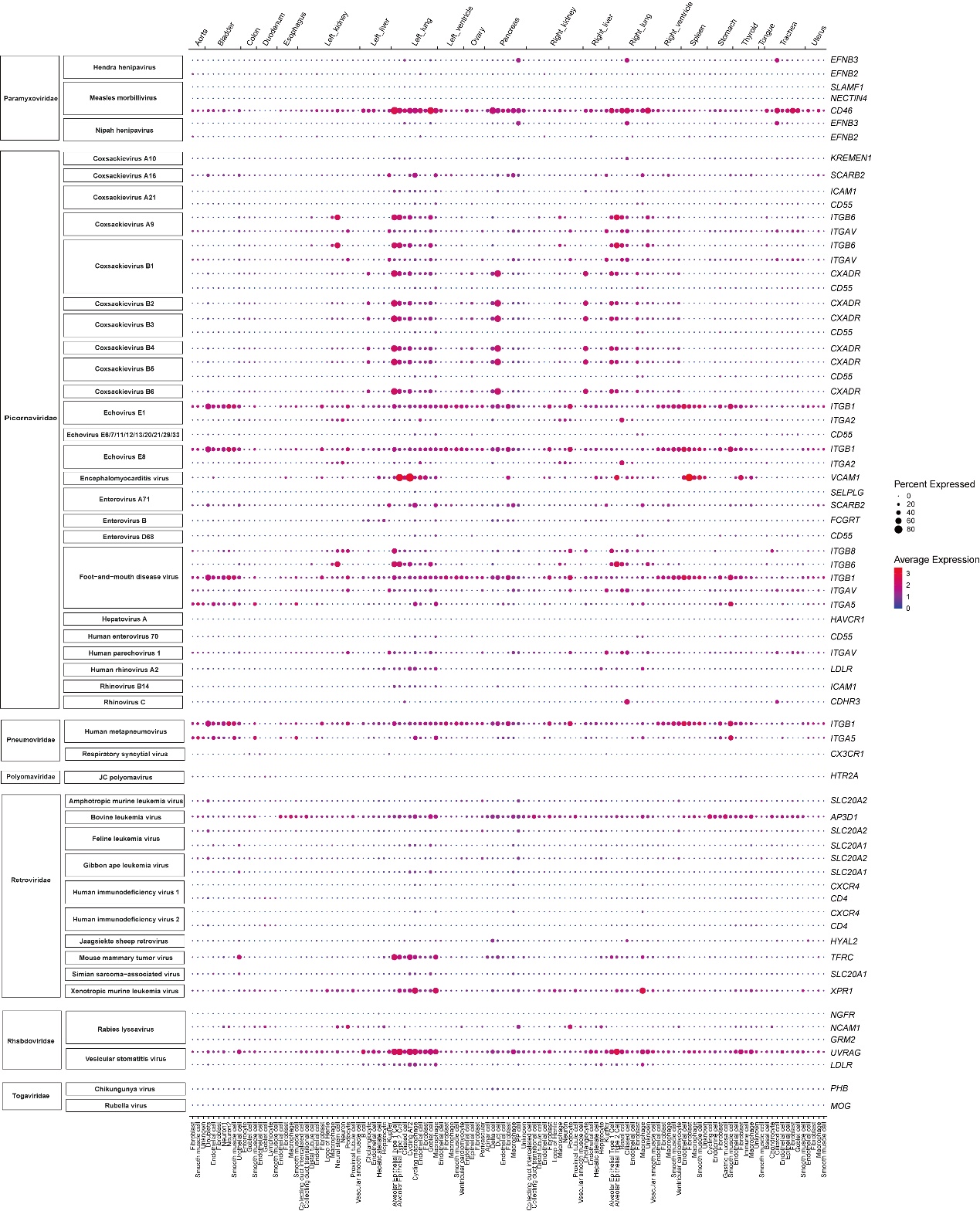


**Fig. S15.** Detection the receptors of other 69 potential viruses for giant panda.
