# Supplementary material for: Single-nucleus transcriptome inventory of giant panda reveals cellular basis for fitness optimization under low metabolism
Source: BMC Biol. 2023 Oct 20;21:222. doi: 10.1186/s12915-023-01691-2 (PMC10588165; doi:10.1186/s12915-023-01691-2)
Supplement: Supplementary file 2 — Additional file 2: Table S1. Sequencing information and output of Cellranger for each library. Table S2. Quality control and cell annotation results of each organ/tissue. Table S3. Information of canonical markers from published literature. Table S4. GO analysis for uncertain cell populations. [file 12915_2023_1691_MOESM2_ESM.docx]

**Table S1.** Sequencing information and output of CellRanger for each library

| **Organ/Tissue** | **Library** | **Number of Reads** | **Mean Reads per Cell** | **Estimated Number of Cells** | **Median UMI Counts per Cell** | **Median Genes per Cell** | **Fraction Reads in Cells (%)** | **Reads Mapped to Genome (%)** | **Reads Mapped Confidently to regions (%)** | | | | |
| --- | --- | --- | --- | --- | --- | --- | --- | --- | --- | --- | --- | --- | --- |
|  |  |  |  |  |  |  |  |  | **Genome** | **Intergenic Regions** | **Exonic Regions** | **Transcriptome** | **Gene Antisense** |
| Aorta | PL2012110005 | 575,674,714 | 530,575 | 1,085 | 144 | 105 | 18.9 | 91.4 | 87.7 | 59.8 | 27.9 | 14.7 | 12.0 |
| Aorta | PL2012110006 | 622,019,949 | 565,987 | 1099 | 143 | 105 | 14.5 | 93.7 | 90.0 | 62.0 | 27.9 | 14.3 | 12.6 |
| Aorta | PL2012110007 | 593,836,033 | 439,552 | 1,351 | 128 | 97 | 15.1 | 93.6 | 89.9 | 62.1 | 27.8 | 14.4 | 12.4 |
| Aorta | PL2012110008 | 636,645,504 | 521,413 | 1,221 | 129 | 99 | 14.3 | 93.4 | 89.6 | 62.1 | 27.6 | 14.2 | 12.5 |
| Bladder | PL2103180013 | 624,730,969 | 118,297 | 5,281 | 888 | 486 | 45.4 | 89.7 | 87.2 | 20.0 | 67.2 | 46.9 | 4.7 |
| Bladder | PL2103180014 | 448,133,949 | 156,854 | 2,857 | 528 | 377 | 48.1 | 95.7 | 93.3 | 25.8 | 67.5 | 50.0 | 5.9 |
| Bladder | PL2103180015 | 621,026,057 | 254,936 | 2,436 | 475 | 344 | 48.1 | 96.0 | 93.8 | 25.4 | 68.3 | 50.9 | 5.4 |
| Bladder | PL2103180016 | 603,904,540 | 286,890 | 2,105 | 452 | 323 | 48.9 | 96.1 | 93.8 | 25.6 | 68.1 | 51.0 | 5.7 |
| Colon | PL2103180009 | 362,639,352 | 95,180 | 3,810 | 84 | 69 | 30.3 | 23.4 | 21.4 | 11.0 | 10.4 | 4.6 | 4.7 |
| Colon | PL2103180010 | 421,246,735 | 159,987 | 2,633 | 343 | 288 | 32.2 | 28.8 | 25.8 | 16.5 | 9.3 | 4.6 | 4.3 |
| Colon | PL2103180011 | 294,612,220 | 118,556 | 2,485 | 255 | 220 | 27.8 | 35.3 | 32.0 | 20.9 | 11.1 | 5.1 | 5.6 |
| Colon | PL2103180012 | 466,422,854 | 194,181 | 2,402 | 361 | 310 | 27.8 | 41.2 | 36.9 | 24.0 | 12.8 | 5.6 | 6.7 |
| Duodenum | PL2103180005 | 474,321,974 | 226,623 | 2,093 | 189 | 175 | 25.9 | 48.1 | 44.4 | 31.3 | 13.0 | 5.4 | 7.3 |
| Duodenum | PL2103180006 | 791,340,985 | 313,526 | 2,524 | 171 | 157 | 24.1 | 68.4 | 64.0 | 44.9 | 19.1 | 8.0 | 10.8 |
| Duodenum | PL2103180007 | 539,856,209 | 261,685 | 2,063 | 278 | 260 | 27.5 | 62.5 | 58.2 | 40.9 | 17.3 | 7.4 | 9.6 |
| Duodenum | PL2103180008 | 635,427,497 | 292,554 | 2,172 | 316 | 286 | 24.9 | 61.5 | 56.6 | 39.9 | 16.7 | 7.1 | 9.2 |
| Esophagus | PL2103180029 | 306,925,135 | 112,303 | 2,733 | 152 | 96 | 27.4 | 51.0 | 49.0 | 32.9 | 16.1 | 8.5 | 6.8 |
| Esophagus | PL2103180030 | 148,265,941 | 56,460 | 2,626 | 130 | 85 | 30.6 | 70.7 | 67.9 | 45.3 | 22.7 | 12.2 | 9.2 |
| Esophagus | PL2103180031 | 181,931,936 | 93,059 | 1,955 | 187 | 115 | 28.3 | 68.7 | 65.8 | 44.4 | 21.5 | 11.3 | 9.1 |
| Esophagus | PL2103180032 | 505,125,652 | 226,513 | 2,230 | 148 | 96 | 26.8 | 87.9 | 84.5 | 57.6 | 27.0 | 13.8 | 11.9 |
| Left lung | PL2012110135 | 563,324,116 | 244,817 | 2,301 | 1,261 | 804 | 36.3 | 71.0 | 68.5 | 29.0 | 39.4 | 27.3 | 8.2 |
| Left lung | PL2012110136 | 748,240,609 | 323,214 | 2,315 | 1,226 | 789 | 37.5 | 87.5 | 84.6 | 36.2 | 48.5 | 33.4 | 10.3 |
| Left lung | PL2012110137 | 827,784,421 | 424,504 | 1,950 | 1,188 | 765 | 37.9 | 89.4 | 86.6 | 36.0 | 50.5 | 35.4 | 9.7 |
| Left lung | PL2012110138 | 814,099,874 | 389,149 | 2,092 | 1,297 | 836 | 39.4 | 83.7 | 81.0 | 31.5 | 49.4 | 34.9 | 9.1 |
| Left kidney | PL2011240275 | 499,432,932 | 341,142 | 1,464 | 554 | 395 | 26.1 | 94.3 | 90.8 | 54.4 | 36.4 | 21.0 | 12.2 |
| Left kidney | PL2011240276 | 718,536,239 | 522,192 | 1,376 | 665 | 460 | 25.0 | 94.8 | 91.4 | 54.9 | 36.5 | 20.7 | 12.5 |
| Left kidney | PL2011240277 | 452,367,988 | 230,917 | 1,959 | 788 | 533 | 28.3 | 88.6 | 85.3 | 50.2 | 35.1 | 20.4 | 11.4 |
| Left kidney | PL2011240278 | 474,625,586 | 191,690 | 2,476 | 385 | 298 | 22.8 | 95.4 | 92.0 | 55.9 | 36.2 | 20.4 | 12.5 |
| Left ventricle | PL2103180045 | 305,188,138 | 56,152 | 5,435 | 608 | 352 | 41.5 | 84.0 | 81.1 | 24.5 | 56.6 | 38.4 | 8.2 |
| Left ventricle | PL2103180046 | 276,957,777 | 47,367 | 5,847 | 688 | 391 | 41.8 | 70.5 | 67.9 | 22.1 | 45.9 | 30.3 | 7.8 |
| Left ventricle | PL2103180047 | 559,368,583 | 96,144 | 5,818 | 724 | 400 | 44.0 | 86.7 | 83.7 | 25.7 | 57.9 | 38.8 | 8.9 |
| Left ventricle | PL2103180048 | 322,380,893 | 62,489 | 5,159 | 723 | 407 | 42.0 | 75.8 | 73.1 | 24.5 | 48.6 | 32.1 | 9.0 |
| Left ventricle | SP2012240837 | 267,860,759 | 77,349 | 3,463 | 406 | 257 | 57.8 | 83.0 | 80.3 | 25.6 | 54.7 | 33.7 | 10.9 |
| Left ventricle | SP2012240838 | 401,634,676 | 181,324 | 2,215 | 531 | 323 | 59.1 | 83.9 | 81.2 | 27.5 | 53.6 | 32.2 | 12.2 |
| Left ventricle | SP2012240839 | 407,310,330 | 200,447 | 2,032 | 750 | 442 | 58.6 | 86.0 | 83.4 | 29.6 | 53.8 | 34.1 | 12.4 |
| Left ventricle | SP2012240840 | 379,800,620 | 114,466 | 3,318 | 792 | 447 | 63.5 | 82.6 | 79.8 | 28.6 | 51.2 | 31.4 | 12.7 |
| Left liver | PL2012110143 | 713,092,472 | 213,436 | 3,341 | 883 | 495 | 28.0 | 82.6 | 79.6 | 29.7 | 49.9 | 33.4 | 10.1 |
| Left liver | PL2012110144 | 440,808,982 | 106,347 | 4,145 | 798 | 458 | 29.3 | 79.6 | 76.5 | 28.8 | 47.7 | 31.6 | 10.5 |
| Left liver | PL2012110145 | 386,698,657 | 115,191 | 3,357 | 781 | 449 | 31.2 | 81.8 | 78.7 | 28.4 | 50.4 | 34.3 | 9.8 |
| Left liver | PL2012110146 | 281,329,051 | 87,723 | 3,207 | 674 | 408 | 28.5 | 73.4 | 70.6 | 27.9 | 42.7 | 28.3 | 9.6 |
| Left liver | PL2103180037 | 265,098,992 | 127,390 | 2,081 | 1,146 | 580 | 42.2 | 88.1 | 84.7 | 25.8 | 58.9 | 42.2 | 6.9 |
| Left liver | PL2103180038 | 542,357,896 | 243,100 | 2,231 | 1,550 | 724 | 45.9 | 89.1 | 86.0 | 27.3 | 58.7 | 41.7 | 7.8 |
| Left liver | PL2103180039 | 266,733,691 | 134,442 | 1,984 | 1,549 | 719 | 44.8 | 88.3 | 85.0 | 27.2 | 57.7 | 41.0 | 7.7 |
| Left liver | PL2103180040 | 253,417,843 | 142,050 | 1,784 | 1,549 | 750 | 42.7 | 69.3 | 66.6 | 21.7 | 44.9 | 31.6 | 6.3 |
| Ovary | SP2012240829 | 308,340,008 | 355,640 | 867 | 587 | 410 | 55.9 | 94.1 | 91.4 | 30.7 | 60.7 | 44.3 | 6.7 |
| Ovary | SP2012240830 | 98,807,700 | 104,117 | 949 | 423 | 305 | 53.0 | 94.2 | 91.6 | 31.5 | 60.1 | 44.2 | 6.7 |
| Ovary | SP2012240831 | 332,549,303 | 354,908 | 937 | 590 | 407 | 53.0 | 93.8 | 91.2 | 31.6 | 59.6 | 43.8 | 6.5 |
| Ovary | SP2012240832 | 683,815,963 | 683,132 | 1,001 | 736 | 496 | 57.3 | 93.0 | 90.1 | 58.7 | 58.7 | 43.3 | 6.6 |
| Pancreas | PL2103180033 | 208,910,272 | 69,221 | 3,018 | 1,315 | 820 | 53.6 | 90.3 | 81.6 | 32.6 | 48.9 | 32.6 | 10.6 |
| Pancreas | PL2103180034 | 432,479,153 | 126,197 | 3,427 | 1,365 | 837 | 52.8 | 94.1 | 84.5 | 34.2 | 50.3 | 33.7 | 10.1 |
| Pancreas | PL2103180035 | 543,601,652 | 147,597 | 3,683 | 1,440 | 876 | 49.4 | 95.1 | 86.5 | 36.4 | 50.2 | 33.3 | 10.7 |
| Pancreas | PL2103180036 | 251,236,113 | 75,696 | 3,319 | 1,260 | 789 | 48.6 | 94.8 | 86.0 | 36.6 | 49.4 | 32.4 | 11.0 |
| Right kidney | PL2011240279 | 711,572,010 | 329,431 | 2,160 | 684 | 473 | 28.4 | 94.1 | 90.7 | 52.7 | 38.0 | 21.7 | 11.8 |
| Right kidney | PL2011240280 | 783,750,025 | 341,503 | 2,295 | 639 | 458 | 25.1 | 93.0 | 89.6 | 54.3 | 35.2 | 19.5 | 12.3 |
| Right kidney | PL2011240281 | 753,185,639 | 351,299 | 2,144 | 649 | 461 | 26.7 | 94.3 | 90.7 | 54.3 | 36.4 | 20.6 | 12.0 |
| Right kidney | PL2011240282 | 754,756,172 | 372,167 | 2,028 | 762 | 527 | 31.0 | 91.9 | 88.6 | 50.1 | 38.5 | 22.6 | 11.0 |
| Right liver | PL2012110147 | 305,214,084 | 85,542 | 3,568 | 1,013 | 629 | 37.0 | 75.5 | 72.8 | 27.0 | 45.8 | 31.4 | 8.9 |
| Right liver | PL2012110148 | 248,669,381 | 81,826 | 3,039 | 847 | 544 | 34.2 | 49.4 | 47.6 | 18.5 | 29.1 | 19.7 | 6.0 |
| Right liver | PL2012110149 | 242,004,047 | 92,509 | 2,616 | 752 | 490 | 34.0 | 31.1 | 30.0 | 11.5 | 18.5 | 12.4 | 3.8 |
| Right liver | PL2012110150 | 247,883,138 | 71,808 | 3,452 | 903 | 572 | 35.0 | 73.6 | 70.9 | 27.4 | 43.6 | 29.8 | 8.6 |
| Right lung | PL2012110139 | 192,927,401 | 283,716 | 680 | 348 | 270 | 24.5 | 7.5 | 7.1 | 3.7 | 3.4 | 2.1 | 1.0 |
| Right lung | PL2012110140 | 776,036,304 | 1,405,862 | 552 | 795 | 545 | 27.3 | 86.0 | 82.6 | 46.7 | 35.9 | 21.2 | 12.1 |
| Right lung | PL2012110141 | 594,126,640 | 991,864 | 599 | 607 | 431 | 29.4 | 84.9 | 81.6 | 44.1 | 37.6 | 22.6 | 11.9 |
| Right lung | PL2012110142 | 388,922,329 | 467,454 | 832 | 586 | 432 | 26.1 | 72.4 | 69.3 | 39.0 | 30.3 | 17.7 | 10.4 |
| Right lung | PL2103180041 | 271,374,467 | 310,852 | 873 | 1,156 | 760 | 46.3 | 91.5 | 88.0 | 43.7 | 44.3 | 29.9 | 11.3 |
| Right lung | PL2103180042 | 309,029,732 | 374,581 | 825 | 1,162 | 767 | 49.3 | 93.1 | 89.7 | 44.1 | 45.7 | 30.9 | 11.6 |
| Right lung | PL2103180043 | 258,167,005 | 363,615 | 710 | 1,295 | 825 | 47.2 | 91.6 | 88.4 | 43.0 | 45.4 | 30.6 | 11.4 |
| Right lung | PL2103180044 | 102,175,994 | 111,181 | 919 | 1,176 | 757 | 47.9 | 76.8 | 74.0 | 35.2 | 38.9 | 26.9 | 9.0 |
| Right ventricle | PL2012300700 | 202,874,957 | 114,553 | 1,771 | 906 | 507 | 61.3 | 52.4 | 50.2 | 17.2 | 33.0 | 20.6 | 7.6 |
| Right ventricle | PL2012300701 | 808,331,728 | 91,929 | 8,793 | 771 | 453 | 71.1 | 73.8 | 70.3 | 21.4 | 48.9 | 29.9 | 11.1 |
| Right ventricle | PL2012300702 | 255,786,143 | 105,305 | 2,429 | 1,062 | 571 | 60.2 | 53.9 | 51.6 | 18.6 | 33.0 | 20.7 | 7.7 |
| Spleen | PL2103180017 | 217,204,278 | 108,115 | 2,009 | 297 | 247 | 41.0 | 21.8 | 19.9 | 9.8 | 10.2 | 7.0 | 2.5 |
| Spleen | PL2103180018 | 319,134,039 | 194,593 | 1,640 | 438 | 346 | 42.2 | 24.8 | 23.0 | 10.9 | 12.1 | 8.3 | 2.9 |
| Spleen | PL2103180019 | 167,254,887 | 98,443 | 1,699 | 195 | 165 | 40.6 | 11.2 | 10.3 | 5.2 | 5.1 | 3.5 | 1.3 |
| Spleen | PL2103180020 | 269,086,834 | 113,778 | 2,365 | 297 | 249 | 41.8 | 34.2 | 31.8 | 16.1 | 15.7 | 10.6 | 4.0 |
| Stomach | PL2103180001 | 148,062,891 | 66,217 | 2,236 | 299 | 196 | 55.0 | 29.4 | 28.1 | 9.2 | 19.0 | 14.6 | 1.8 |
| Stomach | PL2103180002 | 453,605,692 | 233,336 | 1,944 | 1,031 | 593 | 52.5 | 29.8 | 28.0 | 9.8 | 18.3 | 13.7 | 2.7 |
| Stomach | PL2103180003 | 407,031,856 | 549,300 | 741 | 343 | 249 | 43.1 | 95.4 | 92.1 | 50.9 | 41.2 | 26.3 | 11.6 |
| Stomach | PL2103180004 | 523,655,789 | 463,002 | 1,131 | 261 | 198 | 29.9 | 95.7 | 92.4 | 53.5 | 38.9 | 23.2 | 12.3 |
| Thyroid | PL2103180025 | 409,045,715 | 201,302 | 2,032 | 455 | 391 | 31.4 | 48.6 | 44.5 | 26.0 | 18.5 | 10.2 | 7.4 |
| Thyroid | PL2103180026 | 428,897,988 | 122,472 | 3,502 | 433 | 390 | 29.7 | 57.0 | 51.6 | 31.9 | 19.7 | 10.1 | 8.9 |
| Thyroid | PL2103180027 | 240,401,529 | 134,905 | 1,782 | 221 | 201 | 31.3 | 27.8 | 25.4 | 14.9 | 10.5 | 6.0 | 3.9 |
| Thyroid | PL2103180028 | 556,884,870 | 178,089 | 3,127 | 519 | 458 | 30.3 | 65.9 | 60.3 | 36.9 | 23.4 | 12.1 | 10.4 |
| Tongue | PL2103180021 | 162,318,134 | 120,235 | 1,350 | 92 | 81 | 28.3 | 15.4 | 14.0 | 8.7 | 5.3 | 2.9 | 2.1 |
| Tongue | PL2103180022 | 287,302,850 | 159,968 | 1,796 | 196 | 176 | 27.1 | 27.8 | 25.1 | 16.1 | 9.0 | 4.8 | 3.9 |
| Tongue | PL2103180023 | 562,468,716 | 111,822 | 5,030 | 47 | 44 | 33.4 | 93.0 | 89.2 | 61.9 | 27.3 | 12.4 | 14.5 |
| Tongue | PL2103180024 | 588,299,068 | 97,319 | 6,045 | 55 | 51 | 31.7 | 91.9 | 88.2 | 61.6 | 26.6 | 12.3 | 14.0 |
| Trachea | PL2012110001 | 715,747,408 | 406,443 | 1,761 | 880 | 560 | 39.1 | 88.8 | 85.8 | 39.5 | 46.3 | 31.5 | 8.4 |
| Trachea | PL2012110002 | 670,691,927 | 403,060 | 1,664 | 877 | 570 | 42.5 | 90.8 | 87.9 | 38.7 | 49.2 | 33.9 | 8.5 |
| Trachea | PL2012110003 | 664,202,877 | 406,737 | 1,633 | 602 | 412 | 31.7 | 91.7 | 88.9 | 41.7 | 47.2 | 31.8 | 9.3 |
| Trachea | PL2012110004 | 597,295,486 | 351,143 | 1,701 | 783 | 524 | 38.2 | 89.3 | 86.5 | 37.6 | 48.9 | 33.8 | 8.1 |
| Uterus | SP2012240834 | 493,526,071 | 109,770 | 4,496 | 270 | 213 | 64.4 | 97.0 | 94.7 | 27.8 | 66.9 | 49.6 | 7.1 |
| Uterus | SP2012240835 | 557,947,160 | 112,489 | 4,960 | 374 | 298 | 62.1 | 96.5 | 94.1 | 28.7 | 65.3 | 46.2 | 10.2 |
| Uterus | SP2012240836 | 335,853,958 | 72,413 | 4,638 | 318 | 255 | 65.6 | 96.1 | 93.7 | 27.9 | 65.8 | 48.0 | 8.8 |

**Table S2.** Quality control and cell annotation results of each organ/tissue

| **Organ/tissue** | **Raw data** | | | | | | **Filtered data** | | | | | | | |  |  |
| --- | --- | --- | --- | --- | --- | --- | --- | --- | --- | --- | --- | --- | --- | --- | --- | --- |
|  | **Number of nuclei** | **Median UMI** | **Median gene** | **Mean UMI** | **Mean genes** | **Median percent of mitoch-ondrial gene** | **Number of nuclei** | **Median UMI** | | **Median gene** | **Mean UMI** | **Mean genes** | **Median percent of mitoch-ondrial gene** | **Identi-fied cell types** | | **Unknown clusters** |
| Aorta | 4756 | 136 | 101 | 211.52 | 143.45 | 0 | 692 | | 426.5 | 283.5 | 590.6 | 364.54 | 0 | 2 | | 1 |
| Bladder | 12679 | 684 | 419 | 870.83 | 498.63 | 0 | 12010 | | 688 | 422 | 829.5 | 485.82 | 0 | 7 | | 0 |
| Colon | 11330 | 250 | 218 | 337.76 | 269.12 | 1.25 | 6241 | | 359 | 302 | 479 | 382.16 | 1.01 | 3 | | 0 |
| Duodenum | 8852 | 237 | 218 | 339.66 | 289.68 | 0.46 | 5032 | | 327 | 295 | 429.8 | 369.81 | 0.43 | 4 | | 0 |
| Esophagus | 9544 | 150 | 96 | 198.92 | 125.45 | 0 | 1097 | | 402 | 257 | 474.1 | 300.57 | 0 | 4 | | 0 |
| Left kidney | 7275 | 569 | 407 | 838.11 | 531.31 | 0 | 7049 | | 570 | 408 | 811.3 | 522.03 | 0 | 12 | | 0 |
| Left lobe of liver | 22130 | 964 | 521 | 1424.81 | 667.68 | 0.10 | 21429 | | 940 | 511 | 1333 | 644.88 | 0.10 | 6 | | 0 |
| Left lung | 8658 | 1249 | 801 | 1820.38 | 993.23 | 0.04 | 8261 | | 1204 | 777 | 1571 | 909.85 | 0.03 | 10 | | 0 |
| Left ventricle | 33287 | 658 | 378 | 903.67 | 478.97 | 0.15 | 31045 | | 662 | 380 | 898.5 | 479.37 | 0.15 | 5 | | 0 |
| Ovary | 3754 | 576.5 | 402.5 | 880.07 | 543.86 | 0 | 3564 | | 595 | 413 | 875.10 | 547.18 | 0 | 4 | | 0 |
| Pancreas | 13447 | 1346 | 831 | 1557.00 | 906.55 | 0 | 13089 | | 1326 | 820 | 1466 | 870.81 | 0 | 8 | | 0 |
| Right kidney | 8627 | 683 | 480 | 957.01 | 606.45 | 0 | 8494 | | 674.5 | 475 | 912.6 | 587.40 | 0 | 11 | | 0 |
| Right lobe of liver | 12675 | 884 | 564 | 1209.16 | 698.23 | 0 | 12419 | | 870 | 557 | 1157 | 677.23 | 0 | 5 | | 0 |
| Right lung | 5990 | 864 | 594.5 | 1268.09 | 765.70 | 0 | 5694 | | 875 | 602 | 1222 | 753.47 | 0 | 10 | | 0 |
| Right ventricle | 12993 | 833 | 478 | 1118.5 | 575.47 | 0.49 | 12570 | | 811 | 468 | 1074 | 558.2 | 0.48 | 5 | | 0 |
| Spleen | 7713 | 304 | 251 | 428.66 | 332.15 | 0 | 5112 | | 402 | 326 | 536.6 | 411.24 | 0.09 | 4 | | 2 |
| Stomach | 6052 | 477 | 319 | 773.87 | 441.34 | 0 | 3974 | | 744.5 | 457.5 | 977.7 | 561.07 | 0 | 6 | | 0 |
| Thyroid | 10443 | 438 | 390 | 606.14 | 499.49 | 0.26 | 9437 | | 459 | 407 | 611.7 | 511.13 | 0.27 | 4 | | 0 |
| Tongue | 14221 | 60 | 55 | 102.65 | 88.68 | 0 | 920 | | 336.5 | 281 | 436.6 | 353.71 | 0 | 1 | | 2 |
| Trachea | 6759 | 779 | 515 | 1142.81 | 657.82 | 0 | 6651 | | 773 | 510 | 1093 | 641.04 | 0 | 8 | | 0 |
| Uterus | 14094 | 322 | 257 | 419.49 | 315.18 | 0 | 10406 | | 369 | 291 | 456.4 | 343.04 | 0 | 5 | | 0 |

**Table S3.** Information of canonical markers used in this atlas from published literature

|  | **Cell type** | **Abbreviation** | **Exist in organs/tissues** | **Canonical genes** | **Note** |
| --- | --- | --- | --- | --- | --- |
| 1 | Endothelial cell | EC | common | *PECAM1, EMCN, CD34, TM4SF1, AQP1, CD74, LDB2, STAB2, PTPRB, KDR, FLT1, FLI1, VWF, SPARCL1, PCDH17, ADGRL4, EPAS1, CDH5, FABP5* |  |
| 2 | Fibroblast | FB | common | *C7, ABCA6, PDGFRA, DCN, LAMA2, FBLN1, COL6A3, COL6A1, COL3A1, LUM, GSN, COL1A2* |  |
| 3 | Fibroblast_ *ALDH1A2^+^* | FB_*ALDH1A2^+^* | left/right lungs | *ALDH1A2, CCDC80, MGP, C1R, ALDH1A3, SPARC* |  |
| 4 | Smooth muscle cell | SMC | common | *ACTG2, DES, ITGA8, CALD1, ACTA2, TPM2, MYH11, SPARCL1, LPP, MYLK, RGS5, MYL6, ACTB* |  |
| 5 | Macrophage | MP | common | *CD36, IER3, SSBP2, S100A8, MRC1, MSR1, MAMU-DRA, HLA-DQB2, PTPRC, CD14, FCGR2, C1QA* |  |
| 6 | Urothelial cell |  | bladder | *S100P, KLF5, LAMB3, KRT19* |  |
| 7 | Enterocyte |  | colon | *SI* |  |
| 8 | Goblet cell |  | colon, duodenum, left/right lungs, trachea | *FCGBP, CLCA1* (colon); *AGR2* (duodenum); *CXCL17, MUC5B, TFF3, BPIFB1* (lung); *CXCL17, SCGB3A1, WFDC2, MUC5B, FCGBP, AGR2* (trachea) |  |
| 9 | Lymphocyte |  | duodenum | *CD8B, MAML2* |  |
| 10 | Proximal tubule cell | PTC | left/right kidneys | *SLC13A3, SLC34A1, LRP2, CUBN, SLC5A12* |  |
| 11 | Loop of Henle | LOH | left/right kidneys | *UMOD* |  |
| 12 | Distal tubule cell | DTC | left/right kidneys | *SLC12A3, WNK1* |  |
| 13 | Intercalated cell transiting to principal cell | IC-tran-PC | left/right kidneys | *ATP6V0A4, RHCG* |  |
| 14 | Collecting duct intercalated cell – type B | CD-IC-B | left/right kidneys | *SLC26A4, ATP6V0A4, INSRR* |  |
| 15 | Podocyte | POD | left/right kidneys | *PTPRO, PODXL, WT1* |  |
| 16 | Vascular smooth muscle cell |  | left/right kidneys | *CTNNA3, PRKG1* |  |
| 17 | Hepatocyte |  | left/right lobe of livers | *ALB, HP, APOB, FETUB* |  |
| 18 | Hepatic stellate cell | HSC | left/right lobe of livers | *C7, DCN, ACTA2, CXCL12* |  |
| 19 | Cholangiocyte |  | left/right lobe of livers | *PKHD1, CFTR* |  |
| 20 | Kupffer |  | left/right lobe of livers | *CD163, PTPRC, GPNMB, CD5L, MSR1, MARCO, PTPRC, ARHGAP15* |  |
| 21 | B cell |  | left lobe of liver | *JCHAIN, NR4A2* |  |
| 22 | Alveolar epithelial type 1 | AT1 | left/right lungs | *CAV1, CLIC5* |  |
| 23 | Alveolar epithelial type 2 | AT2 | left/right lungs | *LRRK2, SFTPC, ROS1, SFTPB, SFTPA1, SFTPD* |  |
| 24 | Cycling alveolar epithelial type 2 | Cycling AT2 | left lung | *TOP2A, SMC4, CENPF, HMGB2, KIF20B, ATAD2, ROS1, SFTPC* | GO analysis |
| 25 | Cycling macrophage | Cycling MP | left lung | *TOP2A, SMC4, CENPE, CENPF, HMGB2, KIF20B, ATAD2, MSR1, PTPRC, CD14* | GO analysis |
| 26 | Ciliated cell |  | left/right lungs, trachea | *LRRIQ1, CFAP43, HYDIN, CD59, RSPH1, CCDC173* (lung and trachea)*; DNAH12, FHAD1, DNAH7* (trachea) |  |
| 27 | Basal cell |  | right lung, trachea | *TP63, KRT15* (lung and trachea)*; KRT13, KRT4* (trachea) |  |
| 28 | Ventricular cardiomyocyte | vCM | left/right ventricles | *TNNI3K, MYH7B, MLIP, TTN, MYL3, MYL2, TNNI3, MYH7, TNNC1* |  |
| 29 | Perivascular cell |  | ovary | *ACTA2, TAGLN, MYH11, MCAM* |  |
| 30 | Epithelial cell |  | ovary, trachea | *KRT8, KRT18, KRT19* (ovary); *LTF, PIGR, WFDC2* (trachea) |  |
| 31 | Acinar cell |  | pancreas | *CEL, CPA2, NR5A2, XBP1, CPB1, CPA1, CTRB1, GATA4, SMARCA1, CLPS* |  |
| 32 | Duct cell |  | pancreas | *CFTR, KRT8, SPP1, NOTCH2, PKHD1, PPARGC1A* |  |
| 33 | Delta cell |  | pancreas | *RBP4* |  |
| 34 | PP cell |  | pancreas | *PAX6, PTGFR* |  |
| 35 | Endocrine cell |  | stomach | *GAST, CHGA* |  |
| 36 | Pit mucous cell | PMC | stomach | *GKN2, MUC5AC* |  |
| 37 | Gland mucous cell | GMC | stomach | *MUC6* |  |
| 38 | Cycling pit mucous cell | Cycling PMC | stomach | *TOP2A, SMC4, CLSPN, CENPE, CENPF, HMGB2, KIF20B, ATAD2, CBX5* | GO analysis |
| 39 | Chondrocyte |  | trachea | *COL11A1, DCN, ACAN* |  |
| 40 | Immune cell |  | thyroid | *MAMU-DRA, CD74, PTPRC, ARHGAP15* |  |
| 41 | Lymphatic endothelial cell | LEC | uterus | *MMRN1, RELN, LYVE1* |  |
| 42 | Neuron1 |  | bladder | *TH, SNAP25, SCG2* |  |
| 43 | Neuron2 |  | bladder | *SCN7A, CDH19, GPM6B* |  |
| 44 | Neuron |  | left/right kidneys | *CRYAB, NRXN3* |  |
| 45 | Neuron stem cell |  | left kidney | *CRYAB, LGR5* |  |
| 46 | Alpha-like cell |  | pancreas |  | GO analysis |

**Table S4.** GO analysis for uncertain cell populations

| **Cycling AT2 in lungs** | |  |  |  |
| --- | --- | --- | --- | --- |
| **ONTOLOGY** | **ID** | **Description** | **p.adjust** | **Count** |
| BP | GO:0000819 | sister chromatid segregation | 4.23E-16 | 31 |
| BP | GO:0000070 | mitotic sister chromatid segregation | 7.73E-15 | 27 |
| BP | GO:0140014 | mitotic nuclear division | 3.21E-13 | 32 |
| BP | GO:1902850 | microtubule cytoskeleton organization involved in mitosis | 2.40E-11 | 22 |
| BP | GO:0048285 | organelle fission | 3.38E-11 | 38 |
| BP | GO:0008380 | RNA splicing | 5.66E-10 | 34 |
| BP | GO:0033044 | regulation of chromosome organization | 7.55E-08 | 20 |
| BP | GO:0006260 | DNA replication | 6.56E-05 | 19 |
| BP | GO:0016032 | viral process | 0.000135037 | 24 |
| BP | GO:0019058 | viral life cycle | 0.000707664 | 19 |
| BP | GO:0045787 | positive regulation of cell cycle | 0.001800732 | 18 |
|  |  |  |  |  |
| **Cycling MP in left lung** | |  |  |  |
| **ONTOLOGY** | **ID** | **Description** | **p.adjust** | **Count** |
| BP | GO:0000070 | mitotic sister chromatid segregation | 9.41E-07 | 18 |
| BP | GO:0000819 | sister chromatid segregation | 1.80E-06 | 19 |
| BP | GO:0007052 | mitotic spindle organization | 1.95E-06 | 15 |
| BP | GO:0140014 | mitotic nuclear division | 2.40E-06 | 22 |
| BP | GO:0048285 | organelle fission | 7.30E-06 | 28 |
| BP | GO:1902850 | microtubule cytoskeleton organization involved in mitosis | 1.08E-05 | 15 |
| BP | GO:0006898 | receptor-mediated endocytosis | 0.000178661 | 17 |
| BP | GO:0042116 | macrophage activation | 0.000257655 | 11 |
| BP | GO:0002237 | response to molecule of bacterial origin | 0.000316492 | 20 |
| BP | GO:0002495 | antigen processing and presentation of peptide antigen via MHC class II | 0.001572198 | 6 |
| BP | GO:0002263 | cell activation involved in immune response | 0.001904544 | 16 |
|  |  |  |  |  |
| **Cycling PMC in stomach** | |  |  |  |
| **ONTOLOGY** | **ID** | **Description** | **p.adjust** | **Count** |
| BP | GO:0098813 | nuclear chromosome segregation | 8.80E-06 | 12 |
| BP | GO:0000819 | sister chromatid segregation | 2.02E-05 | 10 |
| BP | GO:0006338 | chromatin remodeling | 0.000127169 | 10 |
| BP | GO:0140014 | mitotic nuclear division | 0.001588341 | 9 |
| BP | GO:0048285 | organelle fission | 0.003092216 | 11 |
| BP | GO:0007346 | regulation of mitotic cell cycle | 0.029221119 | 9 |
| BP | GO:0090068 | positive regulation of cell cycle process | 0.043783949 | 6 |
| BP | GO:0000910 | cytokinesis | 0.047027272 | 5 |
| BP | GO:1902099 | regulation of metaphase/anaphase transition of cell cycle | 0.066735141 | 3 |
|  |  |  |  |  |
| **Alpha-like cell in pancreas** | |  |  |  |
| **ONTOLOGY** | **ID** | **Description** | **p.adjust** | **Count** |
| BP | GO:0008380 | RNA splicing | 1.36E-11 | 27 |
| BP | GO:0009896 | positive regulation of catabolic process | 0.013960346 | 14 |
| BP | GO:0005980 | glycogen catabolic process | 0.016588908 | 3 |
| BP | GO:0009251 | glucan catabolic process | 0.019395532 | 3 |
| BP | GO:0044247 | cellular polysaccharide catabolic process | 0.022105795 | 3 |
| BP | GO:0048511 | rhythmic process | 0.059466626 | 9 |
| BP | GO:0032922 | circadian regulation of gene expression | 0.089224085 | 4 |
| BP | GO:0031667 | response to nutrient levels | 0.114586623 | 11 |
| BP | GO:0044275 | cellular carbohydrate catabolic process | 0.116973128 | 3 |
| BP | GO:0042149 | cellular response to glucose starvation | 0.151380462 | 3 |
| BP | GO:1990928 | response to amino acid starvation | 0.155634759 | 3 |
| BP | GO:0032868 | response to insulin | 0.172224048 | 7 |
| BP | GO:0051384 | response to glucocorticoid | 0.173031875 | 5 |
